# Cognitive Behavioral Therapy–Based Digital Interventions for Illicit Substance Use Disorders: Scoping Review of Intervention Design, Efficacy Indicators, and Methodological Gaps

**DOI:** 10.2196/99718

**Published:** 2026-11-03

**Authors:** Sungmin Kim, Su-Ji Jung, Miju Kang, Younghoon Chon, Na-Rae Lee, Hwang Gyu Il, In-Young Choi, Dai Jin Kim, Ji-Won Chun

**Affiliations:** 1 Department of Medical Sciences, College of Medicine, The Catholic University of Korea, Seoul, Republic of Korea; 2 Incheon Chamsarang Hospital, Incheon, Republic of Korea; 3 Department of Psychiatry, Seoul St. Mary’s Hospital, College of Medicine, The Catholic University of Korea, Seoul, Republic of Korea; 4 Department of Medical Informatics, College of Medicine, The Catholic University of Korea, 222 Banpo-daero, Seocho-gu, Seoul, 06591, Republic of Korea, 82 222589727; 5 Institute for Basic Medical Science, The Catholic Medical Center of The Catholic University of Korea, Seoul, Republic of Korea

**Keywords:** substance use disorders, digital health, cognitive behavioral therapy, digital therapeutics, behavioral intervention, app-based intervention

## Abstract

**Background:**

Illicit substance use disorders (SUDs) are escalating globally; yet, social stigma and legal barriers restrict access to conventional treatment, and existing research remains skewed toward alcohol and nicotine. Digital interventions incorporating cognitive behavioral therapy (CBT) offer a discreet, scalable alternative.

**Objective:**

This scoping review maps how CBT-based digital interventions for illicit SUDs are designed and delivered across platforms, identifying their theoretical frameworks, functional components, outcome indicators, and reported methodological limitations.

**Methods:**

We followed the Joanna Briggs Institute (JBI) scoping review methodology and reported in accordance with PRISMA-ScR (Preferred Reporting Items for Systematic Reviews and Meta-Analyses extension for Scoping Reviews). Six databases (PubMed/MEDLINE, Embase, APA PsycINFO, Web of Science Core Collection, Cochrane Library, and CINAHL) were searched from inception to June 28, 2026, without language, date, or study design limits, supplemented by backward and forward citation searching (July 3, 2026). Eligible studies were empirical primary studies quantitatively evaluating digital interventions incorporating CBT for illicit or nonprescribed substance use; alcohol- or nicotine-only populations were excluded. Three reviewers independently screened and charted all studies, with disagreements resolved by consensus or adjudication. Data were synthesized narratively, with tables, figures, and an evidence gap map.

**Results:**

Fifty-six studies published between 2008 and 2026, drawn from 47 distinct participant samples, met the eligibility criteria. Most were conducted in North America (40/56, 71.4%) and used randomized controlled designs (39/56, 69.6%). Polysubstance-using populations were most frequently targeted (32/56, 57.1%), followed by cannabis (13/56, 23.2%), opioids (6/56, 10.7%), and stimulants (5/56, 8.9%). Interventions were delivered mainly via web (24/56, 42.9%) or app (24/56, 42.9%) platforms, and most (49/56, 87.5%) were grounded in traditional CBT, most often combined with contingency management (18/56, 32.1%) or motivational interviewing (17/56, 30.4%). Core functional components included therapeutic intervention (55/56, 98.2%), self-tracking and notification (25/56, 44.6%), reward (24/56, 42.9%), and support and feedback (21/56, 37.5%). Primary outcomes focused on substance use and abstinence (41/56, 73.2%), retention/feasibility (16/56, 28.6%), and mental health status or coping skills (14/56, 25%). Author-reported limitations were predominantly participant-level (52/56, 92.9%), measurement-level (43/56, 76.8%), study-level (34/56, 60.7%), and intervention-level (25/56, 44.6%) domains.

**Conclusions:**

Unlike previous reviews confined to a single substance or delivery platform, this review is innovative in mapping CBT-based digital interventions across the full spectrum of illicit substances, including polysubstance use, and in linking their functional components to intended therapeutic mechanisms. It thereby contributes to the development of these technologies and, by synthesizing author-reported limitations, proposes methodological improvements for their future evaluation. At present, CBT-based digital interventions are best positioned as adjuncts that extend the reach and continuity of existing addiction care rather than as stand-alone substitutes for it; substance-specific customization, objective outcome measures, and modular trial designs are needed to establish them as scalable clinical tools.

## Introduction

### Rationale

Substance use disorders (SUDs) involving illicit drugs, including methamphetamine, cocaine, fentanyl, and cannabis, present a severe global public health crisis. According to the 2025 United Nations Office on Drugs and Crime World Drug Report, an estimated 316 million people worldwide used illicit drugs, reflecting a sharp escalation in synthetic drug markets [1]. In South Korea, which had previously experienced relatively few drug-related problems, the number of drug offenders exceeded 23,000 in 2024, with people in their 20s and 30s accounting for more than 60% of that total [2]. Unlike socially acceptable substances such as alcohol and nicotine, the use of these illicit substances carries profound legal consequences and social stigma, particularly within the strict legal frameworks of East Asia that exacerbate barriers to traditional health care access [3,4]. These systemic obstacles necessitate a distinct and highly accessible treatment approach, yet prior research has often addressed SUDs as a broad category that includes socially acceptable substances such as alcohol and nicotine [5]. Consequently, the existing literature is heavily skewed toward these legal substances, with nicotine-related and alcohol-related studies accounting for 1192 and 1046 publications, respectively [6]. In contrast, research focused on cannabis, opioids, or other illicit drugs remains relatively limited, with only 678 such studies identified [6]. This lack of specialized data is particularly concerning because illicit substances are characterized by strong neurophysiological dependence, rapid tolerance development, severe withdrawal symptoms, and high relapse rates. The frequent association of these substances with impaired self-regulation and co-occurring mental health disorders further complicates treatment interventions, rendering them significantly more complex than those for alcohol- or nicotine-related disorders [7].

Among the emerging approaches in this context, digital interventions have emerged as a promising strategy for the treatment of illicit SUDs. These interventions leverage technologies such as mobile apps, web-based platforms, chatbots, and wearable devices, allowing individuals to engage in treatment autonomously and to be free from time and location constraints [8]. This point is particularly relevant for individuals with SUDs, many of whom avoid in-person care because of stigma, fear of legal consequences, and social marginalization, all of which contribute to high treatment discontinuation rates [9]. Digital interventions offer a discreet and flexible alternative that supports both anonymity and autonomy. Given the high rates of relapse and the need for repeated treatment episodes among members of this population, these interventions may increase acceptability and promote sustained engagement, particularly among individuals who are already familiar with digital tools [10]. In regions where treatment resources are limited or access is restricted, digital interventions provide a scalable and cost-effective solution, highlighting their potential to improve treatment accessibility and effectiveness for SUDs [11,12].

Digital interventions for SUDs exhibit diverse structures and theoretical foundations, although cognitive behavioral therapy (CBT) has served as the predominant framework in many designs. CBT is a widely recognized approach that facilitates behavioral change by addressing irrational thoughts and self-regulatory deficits, making it particularly well-suited for treating SUDs [13]. Its structured techniques, such as identifying craving triggers, applying relapse prevention strategies, and strengthening coping and problem-solving skills, align closely with the clinical needs of this population [14]. In recent years, complementary models, including acceptance and commitment therapy, mindfulness-based therapy (MBT), and dialectical behavior therapy (DBT), have been integrated into intervention designs to address symptom domains such as craving, emotional dysregulation, and impulsivity [15-17]. These approaches provide a broader foundation for the development of tailored digital interventions.

Although digital interventions have shown promising results across various SUDs, existing studies often fail to account for differences in specific substance categories. Given that illicit substances, including stimulants, cannabis, and opioids, differ significantly in their addiction mechanisms and treatment responses, intervention designs must be tailored to distinct user profiles. However, the current literature lacks a comprehensive overview of how digital interventions are applied across these different categories. Previous studies have evaluated digital health interventions targeting specific substances, such as methamphetamine [18] or opioids [19]. However, it is necessary to consider studies that address the use of various substances, including polysubstance use, to evaluate digital interventions in clinical settings. Therefore, a comprehensive review encompassing a wider range of illicit substances is necessary to synthesize how these interventions are designed and adapted across diverse clinical profiles.

Because of the inherent heterogeneity in the design, delivery, and target populations of these interventions, a scoping review provides the most appropriate methodological framework. Accordingly, this study aims to map the current landscape of digital interventions for illicit SUDs, explicitly excluding those related to alcohol and nicotine. Furthermore, by reviewing the efficacy indicators used to validate these interventions and identifying the limitations of each study, this review seeks to provide foundational insights to guide the design of future research.

### Objectives

This scoping review aims to map how CBT-based digital interventions for illicit SUDs are designed and delivered across various platforms. Furthermore, it identifies their common theoretical frameworks and functional components, and collates the efficacy indicators used to validate these interventions. This review also analyzes the specific methodological limitations reported in each study, highlighting the resulting implications for the design of future trials.

## Methods

### Overview

This scoping review followed the framework developed by Arksey and O’Malley [20], as operationalized in the Joanna Briggs Institute (JBI) methodology for scoping reviews, and was reported in accordance with the PRISMA-ScR (Preferred Reporting Items for Systematic Reviews and Meta-Analyses extension for Scoping Reviews) guidelines [21]. The review aimed to comprehensively examine how CBT-based interventions are delivered to individuals with SUDs in digital settings. The PRISMA-ScR checklist is provided in Checklist 1.

### Protocol and Registration

The review was conceived by JWC, YHC, and DJK. A protocol specifying the objectives, questions, eligibility criteria, search strategy, and charting procedures was developed a priori by SMK and SJJ, circulated to all authors, and finalized by consensus before the searches were executed; the charting form developed at this stage is provided in Multimedia Appendix 1. The protocol was not prospectively registered, as PROSPERO does not accept registrations for scoping reviews and no alternative registry was used at the outset; it is available from the corresponding author on request. No departures from the protocol occurred.

### Eligibility Criteria

#### Participants

This review considered studies involving individuals of any age who used illicit or nonprescribed psychoactive substances, or who had a clinically defined SUD related to such substances, as determined by a standardized diagnostic classification (eg, *DSM-5* [*Diagnostic and Statistical Manual of Mental Disorders* {Fifth Edition}] or *ICD-10* [*International Statistical Classification of Diseases, Tenth Revision*]/ICD-11 [*International Statistical Classification of Diseases, 11th Revision*]) or by the criteria applied within each primary study. No age restrictions were applied to capture the full range of populations in which these interventions have been evaluated. Studies involving participants with polysubstance use were eligible if at least one of the substances used was illicit or nonprescribed. Studies were excluded if the population focused solely on alcohol or nicotine/tobacco use, as these substances differ in legal status and are served by separate treatment and evidence traditions, or if the substance of interest could not be determined.

#### Concept

The core concept of interest was the effectiveness of digital interventions grounded in, or incorporating, CBT for the treatment or management of illicit substance use. Digital interventions were defined as any therapeutic program delivered wholly or partly through a digital modality, including web-based platforms, computer programs, smartphone or tablet apps, text messaging (SMS), interactive voice response (IVR), and other mobile- or internet-based technologies, that explicitly applied CBT principles or techniques (eg, cognitive restructuring, coping skills training, and relapse prevention). Eligible studies had to report at least one empirical outcome relevant to intervention effectiveness, such as substance use frequency or quantity, abstinence, craving, or treatment retention/adherence. Studies were excluded if the intervention lacked a digital component, was ungrounded in CBT, did not incorporate CBT, or reported no effectiveness outcome for the digital intervention, including studies reporting usability, feasibility, or acceptability outcomes alone. Eligibility depended on whether such an outcome was reported, not on its direction or magnitude; no study was excluded based on its findings.

#### Context

No restrictions were applied to setting, geographical location, or health system, as the objective of this review was to map the range and contextual applications of these interventions rather than to characterize their effects in any single setting.

#### Types of Evidence Sources

This review considered original, empirical primary studies that quantitatively evaluated the effectiveness of digital interventions incorporating CBT. Eligible designs included randomized controlled trials (RCTs), nonrandomized and quasi-experimental studies, controlled and single-group pre-post studies, observational and cohort studies, and pilot or feasibility studies reporting effectiveness outcomes. This breadth was deliberate: restricting eligibility by design would have excluded much of the evidence this review set out to map. Excluded source types were systematic, scoping, and narrative reviews; study protocols; secondary or descriptive reports presenting no effectiveness outcomes; letters, editorials, commentaries, and errata; and other nonempirical formats. Secondary analyses of previously reported trials were eligible if they were published as independent primary studies and reported their own effectiveness outcomes for a digital intervention. Conference abstracts, dissertations, and other gray literature were ineligible, as such reports seldom describe intervention content, delivery format, and outcome ascertainment in the detail our charting form required.

### Information Sources

Six bibliographic databases were searched from inception to June 28, 2026: PubMed/MEDLINE (National Library of Medicine), Embase (Elsevier), APA PsycINFO (ProQuest), the Web of Science Core Collection (Clarivate), the Cochrane Library (Wiley), and CINAHL (EBSCO).

Following identification of the included studies, citation searching was undertaken on July 3, 2026, as an additional method of identification. Backward citation searching comprised screening the reference lists of all included studies; forward citation searching was conducted using the “cited-reference” function of the Web of Science Core Collection together with the “Cited by” function of Google Scholar. The most recent search was therefore executed on July 3, 2026. No trial registers were searched, and no sources of gray literature were sought, for the reasons given under the “Types of Evidence Sources” section.

### Search

The search strategy applied the JBI 3-step approach in accordance with the JBI Manual for Evidence Synthesis (section 10.2.5). First, a limited search of MEDLINE (PubMed) and CINAHL was conducted to identify articles relevant to the topic. Second, the words appearing in the titles and abstracts of the retrieved records, together with the index terms used to describe them, were analyzed to develop a comprehensive strategy. Three concepts—SUD, CBT, and digital health—were each represented by controlled-vocabulary terms (MeSH in PubMed and the Cochrane Library, Emtree in Embase, APA Thesaurus of Psychological Index Terms in PsycINFO, and CINAHL Subject Headings in CINAHL) combined with a broad set of free-text synonyms, using truncation and the field modifiers appropriate to each platform, with OR within each concept and AND across concepts. The Web of Science Core Collection has no controlled vocabulary, and the strategy for that database therefore used free-text terms alone. The full strategy was then run across all 6 databases with the syntax adapted to each. Third, backward and forward citation searching was performed once the included studies had been identified, as described under the “Information Sources” section.

The strategy was developed by JWC, SMK, SJJ, and MJK, and the searches were executed by SMK, SJJ, and MJK. Before the final searches were run, the strategy was peer-reviewed against the Peer Review of Electronic Search Strategies checklist by 4 authors (YHC, DJK, NRL, and KH) who had not been involved in developing or executing it.

No language, date, publication type, or study design limits or filters were applied in any database. Because the review mapped the extent, range, and nature of the available evidence rather than estimating an intervention effect, restricting retrieval by design would have excluded much of the eligible range; sensitivity was prioritized over precision, consistent with JBI guidance for scoping reviews. For the same reason, the Cochrane Central Register of Controlled Trials was not searched separately, although the Cochrane Library was searched in full. Ineligible source types were removed at screening rather than by database filters so that borderline records were assessed by the reviewers.

The complete strategy for every database—subject headings, free-text terms, field tags, records retrieved at each step, and the date each search was executed—is provided in Multimedia Appendix 2 and reported in accordance with the PRISMA-ScR.

### Selection of Sources of Evidence

Records from all sources were imported into EndNote 21 (Clarivate) and deduplicated by matching on DOI, title, and first author with year, followed by manual verification of all candidate duplicates. Screening decisions were recorded in a shared Microsoft Excel sheet in which each reviewer used a separate sheet so that judgments were made without knowledge of the others’ decisions.

Screening proceeded in 2 sequential stages against the eligibility criteria set out above. First, titles and abstracts were screened to remove clearly ineligible records. Second, the full texts of all potentially eligible records were retrieved and assessed. All records, whether identified through the database searches or through citation searching, were screened at both stages independently by 3 reviewers (SMK, SJJ, and MJK), each of whom assessed every record. Disagreements at either stage were resolved by discussion among the 3 reviewers; where consensus was not reached, the corresponding author (JWC) adjudicated.

Some included reports were found to analyze a dataset shared with one or more other included reports, typically comprising the primary outcome report of a trial together with subsequent secondary analyses, some of which were restricted to a subset of the randomized sample. Such reports were retained as separate sources because each was published as an independent primary study addressing a distinct question and reporting its own effectiveness outcomes, and because excluding them would have removed evidence on intervention components, engagement, and outcomes that the primary reports did not present. Each included publication is accordingly counted as one study throughout this review, and the unit of analysis is the published study rather than the underlying trial.

### Data Charting Process

A draft charting form was developed a priori as part of the protocol and was piloted before full charting began, with SMK, SJJ, and MJK each independently charting the same 10 included studies [22-31]. The pilot confirmed that the prespecified fields captured the required information consistently. All included sources were then charted in full, independently, by the 3 reviewers. Completed forms were compared field by field, and discrepancies were resolved through discussion with the corresponding author (JWC) until consensus was reached. Values reported at only one point in a source and not corroborated elsewhere in the same report were charted as reported and flagged, with no inference beyond the authors’ own statements. Fields for which a source provided no information were charted as “not reported.” The authors of the primary studies were not contacted. The charting form is provided in Multimedia Appendix 1.

### Data Items

Study and participant characteristics comprised the first author, year of publication, country, study design, the target substance and whether polysubstance use was reported, the number of participants analyzed, the proportion who were male, participant age, and intervention period. Engagement characteristics comprised participant retention or completion, completion of the digital intervention, and usage frequency or intensity. Intervention characteristics included the digital delivery mode; theoretical frameworks underpinning the intervention; core functional components—therapeutic intervention (TI), self-tracking and notification (STN), support and feedback (SF), and reward (RW); the name of the digital intervention program; and whether treatment as usual (TAU) was acknowledged in the study. We also extracted the methodological limitations reported by the source authors for each domain.

### Critical Appraisal of Individual Sources of Evidence

No formal critical appraisal of study quality was performed, as this scoping review did not aim to evaluate or compare intervention effectiveness. The focus was on mapping the range, structure, and contextual applications of digital CBT-based interventions. The methodological limitations reported in this review were charted as limitations explicitly acknowledged by the authors of the included studies.

### Synthesis of Results

The charted data were synthesized narratively and presented in tabular and graphical form, including an evidence gap map. Included sources were categorized by delivery mode, intervention theory, core functional components, and the digital intervention program evaluated, and frequencies were tabulated for each category at the level of the included study. Participant retention and intervention adherence were summarized separately, as were the outcome measures used and the reported indicators of effectiveness. The methodological limitations reported by source authors in the included studies were grouped by domain and presented accordingly.

## Results

### Selection of Sources of Evidence

The database searches identified 1676 records: 224 from PubMed, 674 from Embase, 153 from Web of Science, 237 from PsycINFO, 224 from the Cochrane Library, and 164 from CINAHL. Of these, 472 duplicates were removed before screening, leaving 1204 records for title and abstract screening. Screening excluded 1110 records: 644 were nonoriginal articles, 234 did not address substance use, 173 concerned only alcohol or nicotine, and 59 did not concern a digital intervention. All 94 reports sought for retrieval were obtained and assessed at full text, of which 43 were excluded: 22 because no quantitative effectiveness outcome was reported and 21 because only usability or acceptability outcomes were reported. A total of 51 studies were included from the database searches [22-72].

Backward citation searching of the reference lists of these 51 studies identified 2620 records, and forward citation searching identified 3091 records, giving a total of 5711 records. Deduplication removed 1138 records, comprising records duplicated within the citation-searching results together with records already retrieved by the database searches, and left 4573 unique records for title and abstract screening. Screening at this stage excluded 4565 records: 2680 were nonoriginal articles, 950 did not concern substance use, 715 concerned alcohol or nicotine only, and 220 did not concern a digital intervention. All 8 reports sought for retrieval were obtained and assessed at full text, of which 3 were excluded because no quantitative effectiveness outcome for a digital intervention was reported. Five further studies were included from citation searching [73-77].

Across both identification pathways, 5777 unique records were screened at title and abstract, and 102 reports were assessed at full text. In total, 56 studies met the eligibility criteria and were included in the review [22-77], 51 identified through the database searches [22-72] and 5 through citation searching [73-77] (Figure 1). Citation searching thus contributed 5 of the 56 (8.9%) included studies [73-77], which the database searches had not retrieved.

**Figure 1.**
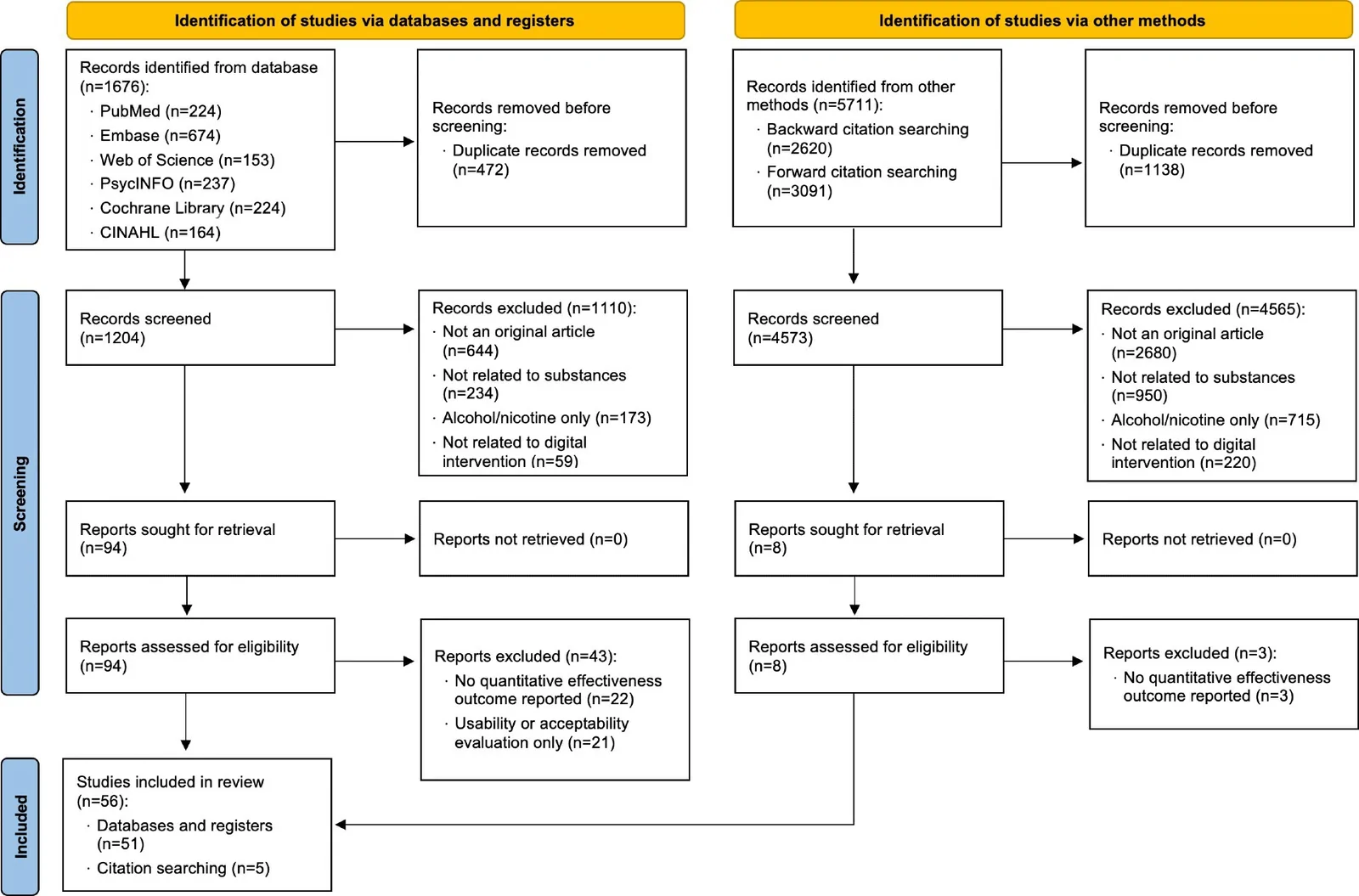
PRISMA (Preferred Reporting Items for Systematic Reviews and Meta-Analyses) 2020 flow diagram of the identification, screening, and selection of sources of evidence for this scoping review of digital interventions incorporating cognitive behavioral therapy for illicit substance use disorder.

Fourteen of the 56 included studies analyzed data drawn from a sample shared with one or more other included studies [24,25,32-43], forming 5 groups. Six studies analyzed data from a single multisite randomized trial conducted in 10 community-based outpatient programs between June 2010 and August 2011 [24,32-36]. Two analyzed a trial conducted in a methadone maintenance program [37,38]. Three further pairs analyzed, respectively, a trial of internet-based DBT [25,39], a trial of a culturally adapted Spanish-language digital CBT program [40,41], and a 3-arm trial of an internet-based self-help program for cannabis use [42,43]. In each group, one study reported the primary outcomes of the parent trial and the remainder reported secondary analyses, several of which were restricted to a subset of the randomized sample. The unit of analysis was the individual published study; the 56 included studies drew on 47 distinct participant samples [22-77].

### Characteristics of Sources of Evidence

Tables 1–3 summarize the key characteristics of the 56 studies included in this review [22-77]. The included studies were published between 2008 and 2026. Among the 56 included studies, the majority were conducted in North America (the United States, Canada, and Mexico), accounting for 71.4% (40/56) [23-27,32-41,44-65,73-75]. The next most common region was Europe, which accounted for 17.9% (10/56) of the studies, including 4 from the United Kingdom, 2 each from Switzerland and the Netherlands, 1 from Sweden, and 1 conducted across Switzerland, Austria, and Germany [22,28,29,42,43,66-70]. East Asia (China and Taiwan) accounted for 5.4% (3/56) [30,71,72], and Australia accounted for 5.4% (3/56) [31,76,77].

**Table 1.** Characteristics of the included studies evaluating cognitive behavioral therapy–based digital interventions for illicit substance use disorders (N=56)^a^^,^^b^.

Author (year)	Country	Target substances	Study design	Participants (male), n (%)	Age, mean (SD)	Intervention period (longest follow-up)
Bickel et al [44] (2008)	United States	Polysubstance (opioid and cocaine)	3-arm RCT^c^	135 (55.7)	28.6 (8.4)	23 weeks (no follow-up)
Carroll et al [73] (2008)	United States	Polysubstance (cocaine, opioid, and cannabis)	2-arm RCT	73 (57.5)	41.6 (10.2)	8 weeks (8 weeks)
Rooke et al [76] (2013)	Australia	Cannabis	2-arm RCT	225 (61.3)	31.0 (9.8)	6 weeks (3 months)
Campbell et al [32] (2014)	United States	Polysubstance (cannabis, cocaine, opioid, and stimulant)	2-arm RCT	507 (62.1)	34.9 (10.9)	12 weeks (6 months)
Marsch et al [37] (2014)	United States	Polysubstance (opioid and cocaine)	2-arm RCT	160 (75)	40.7 (9.8)	12 months (no follow-up)
Budney et al [74] (2015)	United States	Cannabis	3-arm RCT	75 (57)	35.9 (10.5)	12 weeks (9 months)
Campbell et al [33] (2015)	United States	Polysubstance (cannabis, cocaine, opioid, and stimulant)	2-arm RCT	506 (62.1)	34.87 (10.87)	12 weeks (6 months)
Schaub et al [22] (2015)	Switzerland	Cannabis	3-arm RCT	308 (75.3)	29.8 (10.0)	6 weeks (3 months)
Tait et al [77] (2015)	Australia	Stimulant	2-arm RCT	160 (75.6)	22.4 (6.3)	3 weeks (6 months)
Guarino et al [23] (2016)	United States	Opioid	Quasi-experimental study	25 (80)	42.2 (12.5)	12 weeks (no follow-up)
Kim et al [38] (2016)	United States	Polysubstance (opioid and cocaine)	2-arm RCT	160 (75)	40.66 (9.83)	12 months (no follow-up)
Elison et al [66] (2017)	United Kingdom	Polysubstance (cocaine, heroin, cannabis, and stimulant)	Observational study	2311 (55)	42.2 (11.5)	Self-paced (no follow-up)
Kay-Lambkin et al [31] (2017)	Australia	Cannabis	3-arm-RCT	274 (56.9)	40 (NR)^d^	10 weeks (12 months)
Lévesque et al [34] (2017)	United States	Polysubstance (cannabis, cocaine, opioid, and stimulant)	2-arm RCT	437 (62.4)	35.3 (11.0)	12 weeks (6 months)
Liang et al [71] (2018)	China	Polysubstance (heroin and stimulant)	2-arm RCT	74 (70.7)	41.6 (8.0)	4 weeks (1 month)
Paris et al [40] (2018)	United States	Polysubstance (cannabis and cocaine)	2-arm RCT	92 (67.4)	42.9 (11.5)	8 weeks (6 months)
Riggs et al [45] (2018)	United States	Cannabis	2-arm RCT	298 (51)	19.97 (2.0)	Single session (6 weeks)
Tiburcio et al [46] (2018)	Mexico	Polysubstance (cannabis, cocaine, and inhalant)	3-arm RCT	74 (87.8)	NR	8 weeks (1 month)
Marino et al [35] (2019)	United States	Polysubstance (cannabis, cocaine, opioid, and stimulant)	2-arm RCT	507 (62.1)	35.0 (10.9)	12 weeks (6 months)
Moore et al [47] (2019)	United States	Polysubstance (opioid and cocaine)	2-arm RCT	82 (60)	42.4 (10.9)	12 weeks (3 months)
Sinadinovic et al [28] (2020)	Sweden	Cannabis	2-arm RCT	303 (67.3)	27.4 (7.2)	6 weeks (3 months)
Baumgartner et al [42] (2021)	Switzerland, Austria, and Germany	Cannabis	3-arm RCT	575 (70.6)	28.3 (7.9)	6 weeks (3 months)
DeFulio et al [75] (2021)	United States	Opioid	2-arm quasi-experimental	170 (48)	38.0 (9.5)	16 weeks (no follow-up)
Elison-Davies et al [67] (2021)	United Kingdom	Opioid	Observational study	1107 (65.4)	38.42 (9.06)	12 months (no follow-up)
Elison-Davies et al [29] (2021)	United Kingdom	Cannabis	Observational study	1830 (58)	33.8 (11.1)	Self-paced (no follow-up)
Prochaska et al [48] (2021)	United States	Polysubstance (cannabis, opioid, stimulant, and cocaine)	2-arm RCT	180 (35)	40.0 (12.0)	8 weeks (no follow-up)
Prochaska et al [49] (2021)	United States	Polysubstance (cannabis, opioid, stimulant, and cocaine)	Single-group experimental study	101 (24.8)	36.8 (10.0)	8 weeks (no follow-up)
Ahlers et al [43] (2022)	Switzerland	Cannabis	3-arm-RCT	367 (71.6)	27.9 (7.5)	6 weeks (3 months)
Kelpin et al [50] (2022)	United States	Polysubstance (opioid and cocaine)	2-arm RCT	63 (0)	41.2 (12.1)	3.5 weeks (12 weeks)
Luderer et al [36] (2022)	United States	Polysubstance (cannabis, cocaine, and stimulant)	2-arm RCT	206 (64.6)	37.1 (10.87)	12 weeks (no follow-up)
Maricich et al [24] (2022)	United States	Polysubstance (cannabis, cocaine, and stimulant)	2-arm RCT	399 (60.4)	36.3 (10.9)	12 weeks (no follow-up)
Zhang et al [72] (2022)	China	Stimulant/methamphetamine	2-arm RCT	100 (86)	38.2 (7.3)	8 weeks (40 weeks)
Campbell et al [51] (2023)	United States	Polysubstance (stimulant, cannabis, cocaine, and opioid)	2-arm RCT	53 (52.8)	38.6 (10.3)	12 weeks (24 weeks)
Chun-Hung et al [30] (2023)	Taiwan	Stimulant/methamphetamine	2-arm RCT	99 (81.8)	37.0 (10.4)	8 weeks (6 months)
Günther et al [68] (2023)	United Kingdom	Polysubstance (cannabis, cocaine, opioid, and stimulant)	Observational study	12,529 (57.3)	40.3 (11.7)	Self-paced (no follow-up)
Muhlner et al [52] (2023)	United States	Stimulant/methamphetamine	Single-group experimental study	49 (40.8)	41.5 (10.6)	8 weeks (no follow-up)
Olthof et al [69] (2023)	Netherlands	Cannabis	2-arm RCT	378 (69.3)	27.5 (8.5)	6 weeks (6 months)
Paquette et al [26] (2023)	United States	Polysubstance (cocaine, opioid, cannabis, and stimulant)	3-arm RCT	206 (66.5)	40.3 (11.1)	3 weeks (12 months)
Wilde et al [53] (2023)	United States	Polysubstance (unspecified)	Prospective observational study	796 (57.4)	37.7 (NR)	Up to 9 months
Xiong et al [54] (2023)	United States	Polysubstance (cannabis, cocaine, and stimulant)	Observational study	602 (41)	39.0 (11.8)	12 weeks (no follow-up)
Daros et al [25] (2024)	Canada	Polysubstance (cannabis and stimulant)	2-arm RCT	72 (34.7)	34.1 (11.9)	4 weeks (12 weeks)
Garland et al [55] (2024)	United States	Opioid	Single-group experimental study	28 (78.6)	38.5 (9.7)	8 weeks (no follow-up)
Gosens et al [70] (2024)	Netherlands	Polysubstance (cannabis and cocaine)	Single-case experimental design	12 (66.7)	22.9 (4.6)	2‐12 months (3 months)
Benitez et al [41] (2025)	United States	Polysubstance (cannabis and cocaine)	2-arm RCT	85 (68)	43.0 (11.0)	8 weeks (6 months)
Coronado-Montoya et al [56] (2025)	Canada	Cannabis	2-arm RCT	101 (72.3)	25.2 (3.9)	6 weeks (18 weeks)
Daros et al [39] (2025)	Canada	Polysubstance (cannabis and stimulant)	2-arm RCT	72 (34.7)	34.1 (11.5)	4 weeks (12 weeks)
Downie et al [57] (2025)	Canada	Polysubstance (cannabis, stimulant, and opioid)	Single-group experimental study	51 (68.6)	40.6 (12.1)	8 weeks (no follow-up)
Khan et al [58] (2025)	Canada	Polysubstance (cocaine, cannabis, opioid, and stimulant)	Retrospective observational study	3650 (38.9)	NR	Self-paced (no follow-up)
Patel et al [59] (2025)	Canada	Polysubstance (cannabis and cocaine)	2-arm RCT	50 (72)	40.0 (11.4)	8 weeks (6 months)
Stanger et al [60] (2025)	United States	Cannabis	Microrandomized trial	53 (50.9)	22.04 (2.0)	4 weeks (no follow-up)
Stidham et al [61] (2025)	United States	Opioid	Observational study	35 (91.4)	25.1 (2.0)	12 weeks (no follow-up)
Zhang and Hardcastle [62] (2025)	United States	Polysubstance (unspecified)	2-arm quasi-experimental design	193 (50.8)	NR	12 months (no follow-up)
Cardoso Sao Mateus et al [63] (2026)	United States	Stimulant	Retrospective observational study	262 (NR)	41.3 (NR)	16‐26 weeks (no follow-up)
Kawasaki et al [64] (2026)	United States	Opioid	2-arm RCT	100 (49)	36.6 (9.1)	24 weeks (no follow-up)
Prochaska et al [27] (2026)	United States	Polysubstance (cannabis, stimulant, and cocaine)	2-arm RCT	202 (46.5)	38.3 (10.4)	8 weeks (1 month)
Walton et al [65] (2026)	United States	Cannabis	Microrandomized trial	122 (46.7)	21.7 (2.0)	30 days (2 months)

a This scoping review encompasses studies published between 2008 and 2026 across diverse global locations, involving various study designs and targeting diverse clinical populations.

b Studies [32-36] and [24] analyze data from the same parent trial, as do [37] and [38], [40] and [41], [42], and [43], and [25] and [39]. In each group, one study reports the primary outcomes of the trial and the remainder report secondary analyses, several of which were restricted to a subset of the randomized sample. Each was published as an independent primary study and is counted separately; the 56 included studies drew on 47 distinct participant samples.

c RCT: randomized controlled trial.

d NR: not reported.

**Table 2.** Participant retention and intervention adherence metrics across the included studies (N=56)^a^.

Author (year)	Participant retention or completion, n/N (%)	Intervention adherence metrics	
Digital intervention completion	Usage frequency or intensity
Bickel et al [44] (2008)	28/45 (62)	—^b^	70% of scheduled urinalysis sessions attended (69 sessions scheduled)
Carroll et al [73] (2008)	22/35 (62.9)	71.7% (mean 4.3 [SD 2.4]/6 modules)	38.3 (SD 8.2) min/session working with each module
Rooke et al [76] (2013)	64/119 (53.8)	58.3% (mean 3.5/6)	—
Campbell et al [32] (2014)	—	76.3% (mean 36.6 [SD 18.1]/48 modules)	—
Marsch et al [37] (2014)	31/80 (38.7)	52% (mean 27.56 [SD 24.4]/53 modules)	Mean 11.51 (SD 7.21) counselor sessions
Budney et al [74] (2015)	20/30 (66.7)	53.3% (mean 4.8 [SD 3.4]/9 modules)	—
Campbell et al [33] (2015)	—	76.3% (mean 36.6 [SD 18.1]/48 modules)	—
Schaub et al [22] (2015)	79/215 (36.7)	38.3% (pooled mean 3.06/8 modules)	Mean 1.74 d/wk of diary entry (24.9% of days, weeks 1‐6)
Tait et al [77] (2015)	38/81 (46.9)	48.1% (39/81)	—
Guarino et al [23] (2016)	21/25 (84)	92% (23/25)	Mean 2.48 logins/wk
Kim et al [38] (2016)	—	—	—
Elison et al [66] (2017)	1090/2311 (47.2)	27.4% (mean across modules; range 21.6%%‐33.2%)	Median 3 (IQR 7) LBM^c^ strategies completed
Kay-Lambkin et al [31] (2017)	—	—	—
Lévesque et al [34] (2017)	—	—	—
Liang et al [71] (2018)	71/74 (95)	49.3% (mean 13.8 [SD 10.4]/28 daily surveys)	35.4%‐66.7% of participants submitted a daily survey on any given day
Paris et al [40] (2018)	41/43 (95.3)	75.7% (mean 5.3/7 modules)	Mean 4/6 homework assignments completed (66.7%)
Riggs et al [45] (2018)	109/144 (75.7)	100% (single-session design)	Not applicable (single-session intervention)
Tiburcio et al [46] (2018)	9/27 (33.3)	58.3% (mean 4.67 [SD 2.2]/8 sessions)	Mean 5.5 (SD 2.7) strategies per session
Marino et al [35] (2019)	—	76.3% (mean 36.6 [SD 18.1]/48 modules)	—
Moore et al [47] (2019)	32/40 (80)	—	Mean 10.6 (SD 16.1) calls; mean 59.1 (SD 85.6) total minutes of call time
Sinadinovic et al [28] (2020)	50/137 (36.5)	30.0% (mean 3.9 [SD 2.7]/13 modules)	Mean 8.9 visits/wk (mean 62.4 total visits)
Baumgartner et al [42] (2021)	102/386 (26.4)	31.3% (pooled mean 2.50/8 modules)	Pooled mean 42.1 minutes total time on modules
DeFulio et al [75] (2021)	—	66.7% (24/36)	—
Elison-Davies et al [67] (2021)	1107/2571 (43.1)	Mean 4.61 (SD 3.96) BCTs^d^ completed among assessment completers versus 1.07 (SD 1.91) among noncompleters	—
Elison-Davies et al [29] (2021)	460/1830 (25.1)	38.6% (mean 4.63 [SD 4.13]/12 strategies)	Mean 7.55 (SD 10.06) BCTs completed
Prochaska et al [48] (2021)	71/88 (80.7)	30.3% (mean 20 [SD 12.0]/66 lessons)	Mean 4.4 days of use/wk (mean 34.9, SD 28.9, days overall; median 31, IQR 12.0-50.5)
Prochaska et al [49] (2021)	51/128 (39.8)	13.9% completed all psychoeducational lessons; mean 12.1 (SD 8.3) modules	Mean 15.7 (SD 14.2) days of use, equivalent to 2.0 times/week
Ahlers et al [43] (2022)	<30%	28.6% (pooled mean 2.29/8 modules)	Approximately 33% logged in only briefly, completed the first module, and never returned
Kelpin et al [50] (2022)	16/34 (47.1)	71.4% (mean 5 [SD 2]/7 modules)	Mean 4 (SD 3) of 7 weekly homework assignments completed
Luderer et al [36] (2022)	157/206 (76.2)	80.8% (mean 38.8/48 modules)	42.2% (87/206) completed the recommended 4 modules/week throughout
Maricich et al [24] (2022)	157/206 (76.2)	—	Mean 3.3 h/wk of intervention time; 39.8% accessed the program outside the clinic
Zhang et al [72] (2022)	46/52 (88.5)	60.9% (mean 4.87/8 ICBT^e^ sessions)	Mean 2.0 login d/wk (16.37 login days over 8 weeks)
Campbell et al [51] (2023)	21/27 (77.8)	47.5% (mean 22.8 [SD 24.3]/48 modules; median 12.0, IQR 0.0‐48.0)	— (prescribed use: twice weekly, 4 modules/wk)
Chun-Hung et al [30] (2023)	33/50 (66)	75.1% (mean session attendance rate)	Mean retention in treatment 142.42 (SD 60.54) days
Günther et al [68] (2023)	2925/12,529 (23.3)	—	Mean 9 weeks of engagement (SD 17 weeks; range 0 days-142 weeks)
Muhlner et al [52] (2023)	27/49 (55)	>41% of scheduled activities and tasks attended	Mean 55.1 (SD 1.61) days in the 8-week (56-day) program among completers versus 21.9 days among noncompleters
Olthof et al [69] (2023)	126/188 (67)	46.7% (mean 2.8, SD 2.5, of 6 modules)	—
Paquette et al [26] (2023)	52/65 (80)	Mean 4.29 (LETS ACT) and 3.75 (LETS ACT-SE) behavioral activation sessions attended	69% (37/54) reported app use at 1 month after treatment, declining to 37% (20/54) at 3 months
Wilde et al [53] (2023)	350/796 (44)	66.3% (232/350) completed at least 1 BAM survey	Mean 59.4 active days over the 304-day observation window
Xiong et al [54] (2023)	443/602 (74)	52% completed all core lessons	73% completed 4 or more lessons/week in week 1, declining to 36% in week 12
Daros et al [25] (2024)	58/72 (80.6)	35% (24/68) met the recommended dose of 8 days in the first month	Mean 627.47 (SD 576.22) interactions (immediate group, n=38) and 429.17 (SD 560.28; delayed group, n=30)
Garland et al [55] (2024)	23/28 (82)	60.7% (17/28) completed all intervention sessions	—
Gosens et al [70] (2024)	12/21 (57.1)	41.7% (5/12) completed the full protocol	71.2% mean compliance with the daily survey
Benitez et al [41] (2025)	32/39 (82.1)	71.4% (mean 5/7 modules)	—
Coronado-Montoya et al [56] (2025)	30/51 (58.8) completed the core modules	61.7% (mean 3.7, SD 2.5, of 6 modules)	Median total app use 17.8 (IQR 9.7‐30.9) minutes over 6 weeks
Daros et al [39] (2025)	50% active at 4 weeks (94% started the program)	—	Mean 2.5 hours of use over 10 days
Downie et al [57] (2025)	36/51 (70.6)	64.0% (mean 4.48, SD 2.85, of 7 modules)	—
Khan et al [58] (2025)	2295/3650 (62.9)	63% completed at least 1 information or action strategy beyond intake	12% (432/3650) completed strategies at least once at the median dose
Patel et al [59] (2025)	18/25 (72)	—	—
Stanger et al [60] (2025)	43/53 (81.1)	64.6% (4792/7420 EMA^f^ prompts)	Mean 90.28 (SD 32.11) of 140 possible EMA prompts per participant
Stidham et al [61] (2025)	10/35 (28.6) reached 90% or greater lesson engagement	—	—
Zhang and Hardcastle [62] (2025)	69% mean appointment attendance rate (95% CI 65‐74)	—	—
Cardoso Sao Mateus et al [63] (2026)	—	35.9% (pooled mean CBT^g^ module completion)	69.8% (pooled mean survey completion)
Kawasaki et al [64] (2026)	34/48 (70.8)	33.8% (mean 16.2 [SD 13.9]/48 modules)	—
Prochaska et al [27] (2026)	100/107 (93.4)	89% (82/92) completed at least 1 psychoeducational lesson; 51% (47/92) used at least 1 psychotherapeutic tool	Mean 16.2 (SD 13.6) days active
Walton et al [65] (2026)	112/122 (91.8)	77.1% (5642/7320) of twice-daily surveys completed	85.2% (1693/1987) of opened messages actively engaged with

a Data represent user engagement and feasibility outcomes for CBT-based digital interventions targeting illicit substance use disorders.

b Not available.

c LBM: lifestyle balance model.

d BCT: behavior change technique.

e ICBT: internet-based cognitive behavioral therapy.

f EMA: ecological momentary assessment.

g CBT: cognitive behavioral therapy.

**Table 3.** Delivery modes, theoretical foundations, and core functional components of the evaluated digital interventions for illicit substance use disorders (N=56)^a^.

Author and year	Delivery mode	Intervention theory	Intervention components	Digital intervention program	TAU^b^
Bickel et al [44] (2008)	Computer	T-CBT^c^, CM^d^, CRA^e^	TI^f^, SF^g^, RW^h^	Computer-Based CRA	Y
Carroll et al [73] (2008)	Computer	T-CBT	TI	CBT4CBT^i^	Y
Rooke et al [76] (2013)	Web	T-CBT, MI^j^	TI, STN^k^, SF	Reduce Your Use	N
Campbell et al [32] (2014)	Web	T-CBT, CRA, CM	TI, RW	TES^l^	Y
Marsch et al [37] (2014)	Web	T-CBT, CRA	TI	TES	Y
Budney et al [74] (2015)	Web	T-CBT, MI, CM	TI, SF, RW	TES	Y
Campbell et al [33] (2015)	Web	T-CBT, CRA, CM	TI, RW	TES	Y
Schaub et al [22] (2015)	Web	T-CBT, MI	TI, STN, SF	CANreduce2.0	N
Tait et al [77] (2015)	Web	T-CBT, MI	TI, STN	breakingtheice	N
Guarino et al [23] (2016)	App	T-CBT, CRA	TI, RW	TES, Check-In Program	Y
Kim et al [38] (2016)	Web	T-CBT, CRA	TI	TES	Y
Elison et al [66] (2017)	Web and app	T-CBT, MBT^m^	TI	BFO^n^	Y
Kay-Lambkin et al [31] (2017)	Web	T-CBT, MI	TI	SHADE^o^	N
Lévesque et al [34] (2017)	Web	T-CBT, CRA, CM	TI, RW	TES	Y
Liang et al [71] (2018)	App	T-CBT	TI, STN, SF	S-Health	Y
Paris et al [40] (2018)	Web	T-CBT	TI	CBT4CBT	Y
Riggs et al [45] (2018)	Web	PF^p^, PBS^q^	TI, STN	eCHECKUP TO GO	N
Tiburcio et al [46] (2018)	Web	T-CBT	TI, STN, SF	PAADD^r^	Y
Marino et al [35] (2019)	Web	T-CBT, CRA, CM	TI, RW	TES	Y
Moore et al [47] (2019)	Phone (IVR^s^)	T-CBT	TI, STN, SF	The Recovery Line	Y
Sinadinovic et al [28] (2020)	Web	T-CBT, MI	TI, STN, SF	Cannabishjälpen	N
Baumgartner et al [42] (2021)	Web	T-CBT, MI	TI, STN, SF	CANreduce 2.0	Y
DeFulio et al [75] (2021)	App	T-CBT, CM	TI, STN, SF, RW	DynamiCare Health	Y
Elison-Davies et al [67] (2021)	Web and app	T-CBT, MBT	TI	BFO	Y
Elison-Davies et al [29] (2021)	Web and app	T-CBT, MET^t^, MBT	TI	BFO	Y
Prochaska et al [48] (2021)	App (chatbot)	T-CBT, MI, DBT^u^	TI, STN, SF	Weobot (W-SUD)	N
Prochaska et al [49] (2021)	App (chatbot)	T-CBT, MI, DBT	TI, STN, SF	Weobot (W-SUD)	N
Ahlers et al [43] (2022)	Web	T-CBT, MI	TI, STN, SF	CANreduce2.0	N
Kelpin et al [50] (2022)	Web	T-CBT	TI	CBT4CBT	Y
Luderer et al [36] (2022)	App	T-CBT, CRA, CM	TI, RW	TES /reSET	Y
Maricich et al [24] (2022)	Web	T-CBT, CRA, CM	TI, RW	TES	Y
Zhang et al [72] (2022)	App	T-CBT, ApBM^v^, CM	TI, RW	WonderLab Harbor	Y
Campbell et al [51] (2023)	App	T-CBT, CM	TI, RW	TES	Y
Chun-Hung et al [30] (2023)	App (chatbot)	MBT	TI, STN, SF	CAT^w^	Y
Günther et al [68] (2023)	Web and app	T-CBT, MET, MBT	TI	BFO	Y
Muhlner et al [52] (2023)	App	T-CBT, CM, CRA	TI, STN, RW	Affect	N
Olthof et al [69] (2023)	App	T-CBT, MI	TI, STN, SF	iCan	N
Paquette et al [26] (2023)	App	BT^x^	TI	LETS ACT	Y
Wilde et al [53] (2023)	App	T-CBT, CM	TI, STN, SF, RW	Connections	Y
Xiong et al [54] (2023)	App	T-CBT, CRA, CM	TI, STN, RW	reSET	Y
Daros et al [25] (2024)	Web	DBT	TI, SF, RW	Pocket Skills 2.0	N
Garland et al [55] (2024)	App (VR)	MBT, T-CBT	TI	MORE-VR^y^	Y
Gosens et al [70] (2024)	App	T-CBT, MI	TI, STN, RW	Take it Personal!	N
Benitez et al [41] (2025)	Web	T-CBT	TI	CBT4CBT	Y
Coronado-Montoya et al [56] (2025)	App	BT, MI	TI, SF	CHAMPS^z^	Y
Daros et al [39] (2025)	Web	DBT	TI, SF, RW	Pocket Skills 2.0	N
Downie, et al [57] (2025)	Web	T-CBT	TI	CBT4CBT	Y
Khan et al [58] (2025)	Web and app	T-CBT, MET, MBT	TI	BFO	N
Patel et al [59] (2025)	Web	T-CBT	TI	CBT4CBT	Y
Stanger et al [60] (2025)	App	MBT, T-CBT	TI, STN, RW	MetricWire	N
Stidham et al [61] (2025)	App	T-CBT, CM	TI, RW	reSET	Y
Zhang and Hardcastle [62] (2025)	App	CM	STN, RW	DynamiCare	Y
Cardoso Sao Mateus et al [63] (2026)	App	T-CBT, CM	TI, STN, RW	DynamiCare	Y
Kawasaki et al [64] (2026)	App	T-CBT, CM	TI, STN, RW	reSET-O	Y
Prochaska et al [27] (2026)	App (chatbot)	T-CBT, MI, DBT	TI, STN, SF	W-SUD (Woebot)	N
Walton et al [65] (2026)	App	T-CBT, JITAI^aa^, RL^ab^	TI, STN, SF, RW	MiWaves	N

a The evaluated programs are categorized by their primary digital delivery platform, adjunctive theoretical frameworks, and specific interactive elements.

b TAU: treatment as usual.

c T-CBT: traditional cognitive behavioral therapy.

d CM: contingency management.

e CRA: community reinforcement approach.

f TI: therapeutic intervention.

g SF: support and feedback.

h RW: reward.

i CBT4CBT: Computer-Based Training for Cognitive Behavioral Therapy.

j MI: motivational interviewing.

k STN: self-tracking and notification.

l TES: therapeutic education system.

m MBT: mindfulness-based therapy.

n BFO: Breaking Free Online.

o SHADE: self-help for alcohol/other drugs and depression.

p PF: personalized feedback.

q PBS: protective behavioral strategies.

r PAADD: programa de ayuda para abuso de drogas y depresión.

s IVR: interactive voice response.

t MET: motivational enhancement therapy.

u DBT: dialectical behavior therapy.

v ApBM: approach bias modification.

w CAT: chatbot-assisted therapy.

x BT: behavioral therapy.

y MORE-VR: virtual reality-based mindfulness-oriented recovery enhancement.

z CHAMPS: Cannabis Harm-reducing App to Manage Practices Safely.

aa JITAI: just-in-time adaptive intervention.

ab RL: reinforcement learning.

With respect to the targeted substance groups, each study was categorized into 1 of 4 distinct primary categories based on its therapeutic focus (polysubstance, cannabis, opioids, or stimulants). The most frequently targeted substance group was individuals with polysubstance use, which was represented in 57.1% (32/56) of the studies [24-27,32-41,44,46-51,53,54,57-59,62,66,68,70,71,73]. Because this polysubstance category encompasses highly heterogeneous clinical profiles, we further examined the predominant illicit substance patterns within these studies. When excluding alcohol to focus strictly on illicit use, 3 major profiles emerged: first, an opioid and cocaine co-use profile primarily seen in medication-assisted treatment settings [38,44,47,50,71]; second, a cannabis-dominant profile commonly co-occurring with cocaine or stimulant use [24,25,27,36,39-41,46,54,59,70]; and third, a broadly mixed illicit profile with relatively equal proportions of cannabis, cocaine, opioids, and stimulants [26,32-35,48,49,51,57,58,66,68,73].

This group followed by cannabis users in 23.2% (13/56) of the studies [22,28,29,31,42,43,45,56,60,65,69,74,76], opioid users in 10.7% (6/56) [23,55,61,64,67,75], and stimulant users in 8.9% (5/56) [30,52,63,72,77]. Most stimulant-related studies focused on individuals using methamphetamine. No studies targeting hallucinogen use as the primary focus were identified.

### Retention and Adherence Metrics

To evaluate the feasibility and real-world scalability of the included studies, participant retention and intervention adherence metrics were extracted (Table 2). Among the 56 included studies [22-77], 85.7% (48/56) reported participant retention or protocol completion rates during the study period [22-30,36,37,39-62,64-74,76,77]. Reported retention rates showed a wide variance depending on the study design and environment, ranging from the lowest rates of 23.3% [68], up to the highest rates of 95.3% [40]. Conversely, 14.3% (8/56) of the studies did not explicitly report participant retention rates [31-35,38,63,75].

Intervention adherence was primarily measured through digital intervention completion rates and usage frequency or intensity. Digital intervention completion rates were reported in 80.4% (45/56) of the studies [22,23,25-30,32,33,35-37,40-43,45,46,48-58,60,63-67,69-77], while 19.6% (11/56) did not report specific completion metrics [24,31,34,38,39,44,47,59,61,62,68]. The completion rates varied significantly, reaching 100% in a single-session design [45], whereas other interventions observed much lower completion rates, such as 13.9% [49].

In addition, engagement indicators varied widely and fell into 3 groups: time-based metrics, such as minutes per session [73], total time on modules [42], total app use [56], or intervention hours per week [24]; frequency of use, such as logins or days of use per week [23,48,49,72], total active days [52,53], or total program visits [28]; and completion of therapeutic tasks, such as the number of CBT strategies or behavior change techniques completed [29,58,66,67], weekly homework assignments [40,50], or in-platform interactions [25,39].

### Intervention Program Characteristics

#### Delivery Mode

Digital interventions were categorized on the basis of their delivery modalities into web-based, app-based, combined web and app formats, computer-based, phone-based, and technology-assisted formats such as virtual reality (Figure 2A). Among the 56 included studies [22-77], 42.9% (24/56) evaluated interventions accessible directly through web browsers without the need to install separate apps [22,24,25,28,31-35,37-43,45,46,50,57,59,74,76,77]. An additional 8.9% (5/56) of the studies provided interventions that were accessible via both web and app platforms [29,58,66-68]. Several of these web-based and combined interventions incorporated self-directed learning modules, text and video-based content, and self-monitoring tools such as consumption diaries. The structure was often designed to guide users through scheduled content and assigned tasks at regular intervals. Additionally, 3.6% (2/56) used a computer-based delivery mode [44,73], and 1.8% (1/56) used a phone-based IVR system to deliver the intervention [47].

**Figure 2.**
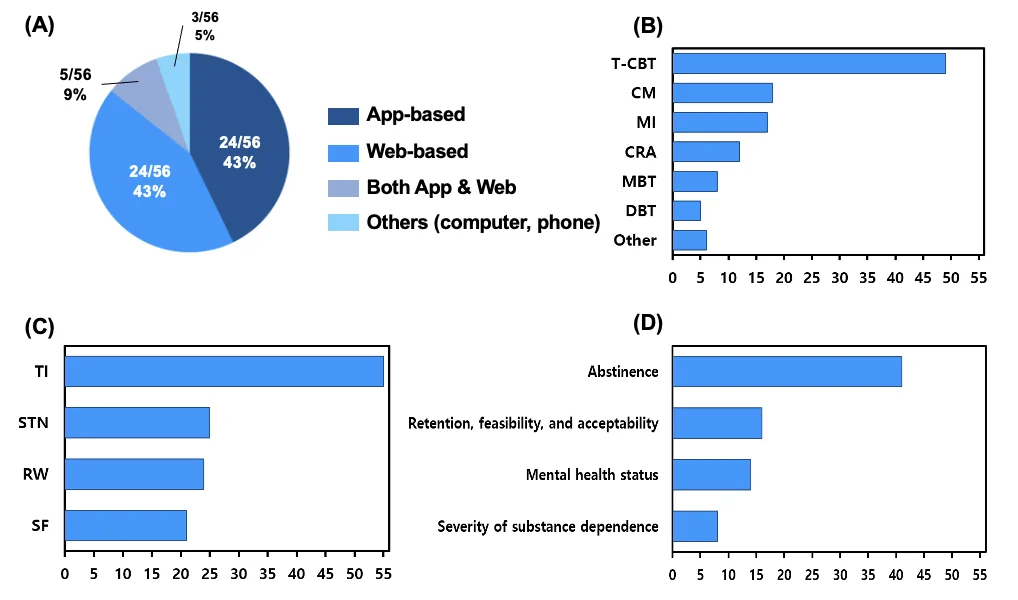
Comprehensive mapping of cognitive behavioral therapy–based digital interventions for illicit substance use disorders (N=56). The figure illustrates the proportional distribution of (A) delivery modes, (B) theoretical foundations, (C) functional intervention components, and (D) primary efficacy outcomes across the reviewed literature. CM: contingency management; CRA: community reinforcement approach; DBT: dialectical behavior therapy; MBT: mindfulness-based therapies; MI: motivational interviewing; RW: reward; SF: support and feedback; STN: self-tracking and notification; T-CBT: traditional cognitive behavioral therapy; TI: therapeutic intervention.

App-based interventions were the most frequently used delivery mode, representing 42.9% (24/56) of the studies, and were accessible via smartphones or tablets [23,26,27,30,36,48,49,51-56,60-65,69-72,75]. Within this context, 4 studies implemented interventions primarily through automated, interactive chatbot systems (eg, Woebot and CAT) [27,30,48,49]. Several app-based interventions included features such as real-time monitoring, push notifications, RW systems, and chatbot-based feedback to enhance user engagement and promote self-regulation. For instance, interventions such as the reSET app (Pear Therapeutics) used a contingency management (CM) structure in which RWs were provided on the basis of the completion of CBT tasks and negative urinalysis results, thereby improving user adherence and participation [61]. The CAT app, which was delivered via the LINE messaging platform (LY Corporation), offered interactive features, including emotional feedback, treatment schedule reminders, daily mood check-ins, and automated responses tailored to user input [30].

In some of the earliest digital intervention studies (3/56, 5.4%), computer-assisted therapy programs were used to provide fluency-based instruction and CM based on the community reinforcement approach (CRA) [44,73,74]. Another study (1/56, 1.8%) used a phone-based IVR system to deliver automated, self-management CBT modules, which offered immediate therapeutic availability 24 hours a day [47]. More recently, in one study (1/56, 1.8%), a virtual reality–based app intervention (MORE-VR; RealizedCare) was implemented to provide an immersive environment for exposure to addiction-related cues and training in coping strategies [55]. The intervention allowed for pre- and postsession assessments of emotional response and craving intensity while tracking physiological data such as heart rate during virtual cue exposure [55].

#### Intervention Theory

Among the 56 studies included in this review [22-77], 87.5% (49/56) used digital interventions grounded in traditional cognitive behavioral therapy (T-CBT) [22-24,27-29,31-38,40-44,46-55,57-61,63-77] (Figure 2B). T-CBT–based interventions typically involve standardized techniques such as cognitive restructuring, coping skills training, and psychoeducation, and are implemented via programs such as Computer-Based Training for Cognitive Behavioral Therapy (CBT4CBT) [40,41,46,50,57,59,73], reSET [24,36,54,61,64], and the Therapeutic Education System [23,32-35,37,38,51]. Among the 49 studies that used T-CBT [22-24,27-29,31-38,40-44,46-55,57-61,63-77], 41 integrated additional theoretical frameworks alongside T-CBT [22-24,27-29,31-38,42-44,46,48,49,51-55,58,60,61,63-70,72,74-77]. CM emerged as the second most commonly used framework, applied in 32.1% (18/56) of the studies [32-36,44,51-54,61-64,72,74,75]. The integration of CM aimed to strengthen user engagement by providing structured RWs based on behavioral targets, such as session completion or abstinence verification. Additionally, the CRA was frequently used alongside T-CBT and CM, appearing in 21.4% (12/56) of the studies [23,24,32-38,44,52,54].

Motivational interviewing (MI), including motivational enhancement therapy, was applied in 30.4% (17/56) of the studies [22,27-29,31,42,43,48,49,56,58,68-70,74,76,77]. MI is frequently used in conjunction with T-CBT to enhance intrinsic motivation and facilitate behavioral change, particularly during the early phases of treatment engagement. In multitheoretical interventions, distinct therapeutic frameworks have been applied to specific functional components. For instance, coping strategies were delivered via CBT modules, whereas motivation enhancement was implemented through MI techniques.

Emotion-focused approaches were also adopted in a subset of the studies. MBT was applied in 14.3% (8/56) of the interventions [29,30,55,58,60,66-68], whereas DBT was used in 8.9% (5/56) [25,27,39,48,49]. In 2 of the DBT-based studies, the intervention program Pocket Skills 2.0 was implemented exclusively on the basis of DBT principles and focused on training emotion regulation and self-awareness skills [25,39].

A small number of studies used alternative approaches. One study (1/56, 1.8%) used a digital CM approach, which aimed to reinforce substance abstinence and treatment adherence by providing monetary incentives via a smartphone app [62]. Furthermore, other emerging frameworks, such as behavioral therapy without cognitive components, the transtheoretical model, personalized normative feedback, and reinforcement learning, were each explored in a minority of studies [26,45,46,56,65].

#### Intervention Components

Almost all included studies (55/56, 98.2%) incorporated a core TI, which was typically delivered through learning modules, psychoeducational videos, problem-solving exercises, and coping skills training [22-61,63-77] (Figure 2C). STN features were used in 44.6% (25/56) of the studies to encourage user engagement by facilitating regular tracking of substance use patterns, mood states, and cravings, as well as through real-time notifications [22,27,28,30,42,43,45-49,52-54,60,62-65,69-71,75-77]. In some cases, users completed remote biological drug testing, such as self-administered saliva tests under virtual supervision, with the results uploaded and tracked via mobile apps. These activities represented an extended form of behavioral self-monitoring [52,56,75].

RW systems were implemented in 42.9% (24/56) of the studies, and monetary or virtual incentives were offered in return for specific achievements such as maintaining abstinence, completing tasks, or logging self-monitoring data [23-25,32-36,39,44,51-54,60-65,70,72,74,75]. In CM-based interventions, the RW component functioned as a key motivational driver to encourage sustained app use. SF components were provided in 37.5% (21/56) of the studies via in-app messaging, automated responses, chatbot interfaces, and live feedback from counselors [22,25,27,28,30,39,42-44,46-49,53,56,65,69,71,74-76]. These features were designed to support users as they progressed through educational content and tasks.

A total of 71.4% (40/56) of the studies integrated combinations of 2 or more of these components [22-25,27,28,30,32-36,39,42-49,51-54,56,60-65,69-72,74-77]. Several studies incorporated gamified features or task-based formats (eg, earning points, unlocking content, or winning accessories for avatars) to enhance user engagement [25,39,70]. Furthermore, some interventions were personalized on the basis of user characteristics, including specific personality traits, levels of functional impairment, risk classification, and baseline assessment results [29,58,66-68,70].

To address the theoretical linkage between the functional components and their presumed mechanisms of action, Figure 3 presents a conceptual framework mapping each component to its hypothesized mechanism and to the outcomes it is intended to affect. TI was present in almost all included studies (55/56) and operates through cognitive restructuring and coping-skills training, targeting drug-related expectancies and craving [22-61,63-77]. STN (25/56) works through self-monitoring and functional analysis, intended to increase behavioral awareness and the recognition of high-risk situations and thereby self-efficacy [22,27,28,30,42,43,45-49,52-54,60,62-65,69-71,75-77]. SF (21/56) draws on supportive accountability and the therapeutic alliance [22,25,27,28,30,39,42-44,46-49,53,56,65,69,71,74-76]; these are mechanisms of engagement rather than of cognitive or behavioral change, and the outcomes they target are retention and continued use of the intervention rather than substance use itself [22,42,49]. RW (24/56) is grounded in operant conditioning and functions as digital CM [23-25,32-36,44,51-54,60-65,70,72-75], reinforcing verified behavioral targets such as drug-negative urinalysis [32,44,75]. CM was named as an adjunctive framework in 18 of the 56 studies [32-36,44,51-54,61-64,72,74,75]. The mechanisms shown are those hypothesized in the source reports; none of the included studies tested them directly.

**Figure 3.**
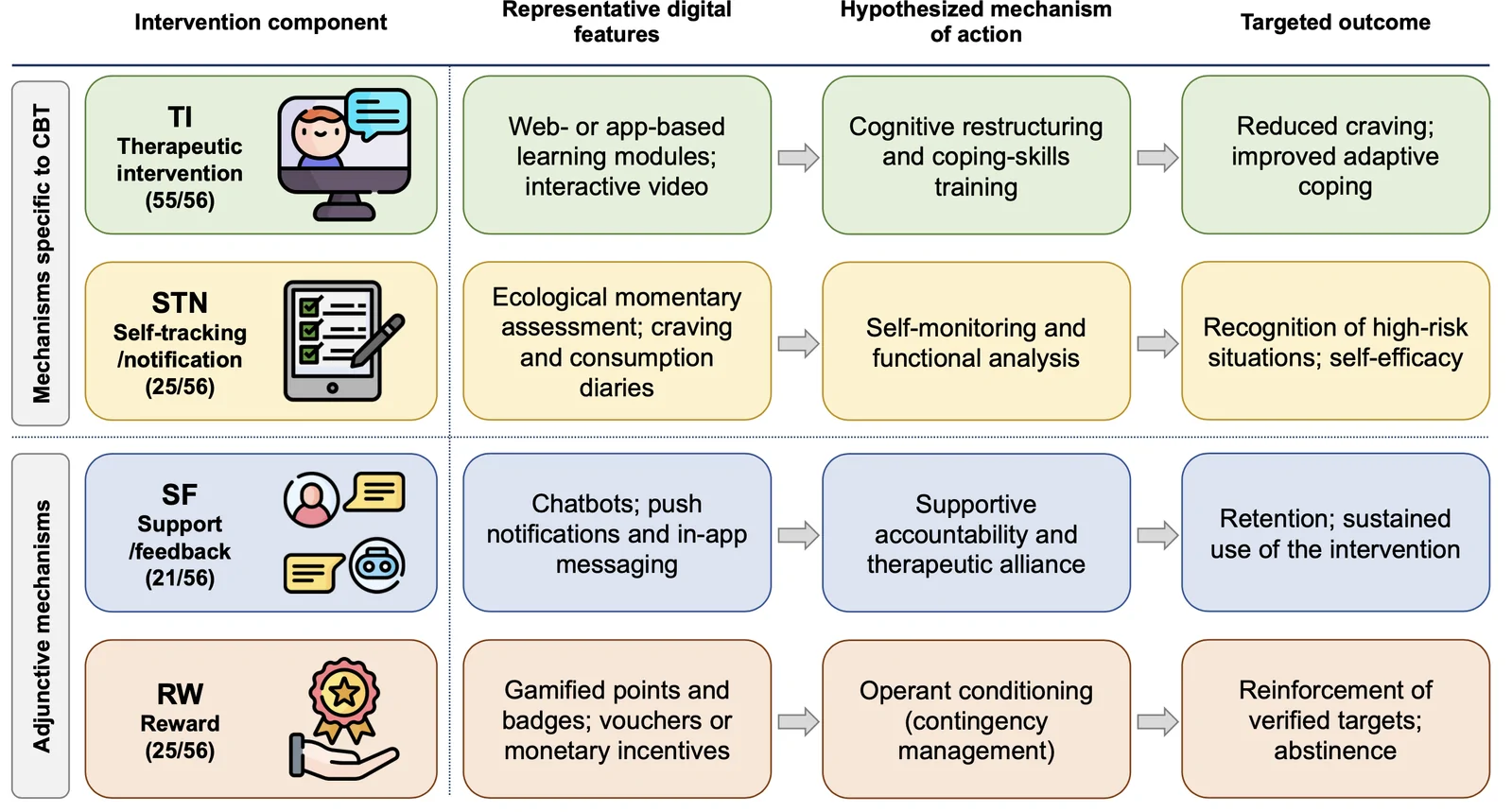
Conceptual framework mapping the digital intervention components identified in this review to their hypothesized mechanisms of action and targeted outcomes. Figures in parentheses are the number of included studies (out of 56) in which each component was identified. The upper 2 components operate through mechanisms specific to cognitive behavioral therapy; the lower 2 operate through adjunctive engagement and operant mechanisms that are frequently combined with cognitive behavioral therapy in digital interventions. CBT: cognitive behavioral therapy; RW: reward; SF: support and feedback; STN: self-tracking and notification; TI: therapeutic intervention.

### Research Methodology

#### Study Design

A total of 69.6% (39/56) of the studies used an RCT design to evaluate the effectiveness of digital intervention programs [22,24,26-28,30-48,50,51,54,56,59,60,64,65,69,71-74,76,77]. Specifically, 29 studies adopted a 2-arm RCT design [24,25,27,28,30,32-41,45,47,48,50,51,56,59,64,69,71-73,76,77], 8 studies used a 3-arm RCT design [22,26,31,42-44,46,74], and 2 studies used a microrandomized trial design [60,65]. Three studies used a quasi-experimental design [23,62,75]. Additionally, 5 studies used a single-group or single-case experimental design [49,52,55,57,70], while the remaining 16.1% (9/56) adopted observational study designs [29,53,54,58,61,63,66-68].

The control conditions (Multimedia Appendix 3) [23,24,26,29,30,32-38,40-42,44,46,47,50,51,53-57,59,61-64,66-68,71-75] were categorized into those with and without active comparators. Some control groups received active interventions such as standard pharmacotherapy, in-person psychotherapy, or active educational modules [23,24,26,27,30-38,40,41,44-47,50,51,56,59,62,64,69,71-76]. In contrast, control conditions without active components primarily used waitlist or delayed-intervention formats [22,25,28,39,42,43,48,77]. Some studies included both active and waitlist control arms, and others used control messages [60,65]. Observational studies and single-group experimental designs did not include formal control groups. Furthermore, TAU or usual care was explicitly integrated into the intervention design in 64.3% (36/56) of the studies [23,24,26,29,30,32-38,40-42,44,46,47,50,51,53-55,59,61-64,66-68,70-73,75], whereas the remaining studies did not specify this component.

The duration of interventions with fixed schedules ranged from a single session to 52 weeks, with a mean duration of 13.7 weeks. Four studies [29,58,66,68] offered self-paced interventions without fixed end points. Among the 32 studies reporting follow-up data [22,25-28,30-35,39-43,45-47,50,51,56,59,65,69-74,76,77], periods ranged from 4 to 52 weeks, with an average of 20.2 (SD 12.6) weeks. Across the studies reporting demographic data, the mean age of participants was 35.3 (SD 6.5) years, and the average proportion of male participants was 56.3% (17,693/31,422).

#### Primary Outcome

The studies included in this review used a range of indicators to assess the effectiveness of digital interventions. The primary outcome measures reported across studies can be broadly categorized into the following 4 domains (Figure 2D).

Changes in substance use behavior and abstinence were the most frequently assessed outcomes, evaluated as a primary measure in 73.2% (41/56) of the studies [22-24,26-34,36-38,40,42-52,54,59,62,63,65,67,69-71,73-77]. Most studies used self-reported data to measure the frequency, number of use days, or quantity of substance use within the past 7 or 30 days [22,24,27-29,31,40,45,47-49,52,67,73,77]. The timeline follow-back method was frequently used as a standardized self-report tool [26,32-34,36,42,43,46,50,51,59,69,71,74,76], while some studies used ecological momentary assessment (EMA) via a mobile app to track daily use quantities [65,70]. To complement self-reported data, several studies incorporated biological verification, including urine tests, saliva tests, and breathalyzers [24,32-34,36,40,47,50-52,71,73,74]. Although most studies reported positive outcomes, some studies found no significant differences or reductions in use between the digital intervention and control groups [28,51,59,69,77].

Treatment retention, feasibility, and acceptability were examined as primary outcomes in 28.6% (16/56) of the studies [24,30,32,33,37,52,54-56,61-65,74,75]. Treatment retention and adherence were assessed using indicators such as time to last contact, clinic appointment attendance, and continued engagement in medication for opioid use disorder programs based on prescription data or electronic health records [24,30,32,33,37,52,54,61-64,74,75]. In several of these studies, digital interventions coupled with CM or app-based monitoring were associated with higher treatment retention and attendance compared to standard care [24,30,52,54,61-63,75]. Other studies explicitly focused on the feasibility and acceptability of the digital platforms, measuring completion rates, ease of use, satisfaction, program engagement via in-app logs, and the presence of adverse events [55,56,65]. These studies reported high usability, safety, and acceptance among the users studied.

A total of 25% (14/56) of the included studies designated mental health status, craving, or coping skills as their primary outcomes [29,31,34,35,41,45,46,49,53,58,60,66,68,72]. Common psychiatric outcome measures included symptoms of depression and anxiety, and overall quality of life [29,31,35,46,49,58,66,68]. Cue-induced craving was evaluated using visual analog scales or EMA to assess momentary emotional reactivity and impulse control [49,60,72]. Additionally, adaptive coping strategies and protective factors were measured using specific tools such as the Drug Risk Response Test and the Brief Addiction Monitor [34,41,45,53]. Most studies in this category reported that digital interventions were associated with reductions in psychiatric symptoms and craving, and with improved coping skills.

Severity of substance dependence and recovery progression were identified as primary efficacy outcomes in 14.3% (8/56) of the studies [25,29,39,46,57,58,66,68]. Most of these studies used the Severity of Dependence Scale to evaluate the intervention’s impact on psychological dependence [25,29,39,57,58,66,68]. Furthermore, to evaluate multifaceted recovery across physical, psychological, and social domains, the Recovery Progression Measure was used to track reductions in functional impairment [29,58,66,68]. These studies generally reported reductions in dependence severity and improvements in recovery progression among users of the digital interventions.

#### Study Limitations

To provide foundational insights for the design of future trials, this review analyzed the methodological limitations explicitly acknowledged by the authors of the included studies. Each reported limitation was assigned to 1 of 4 mutually exclusive domains consisting of study-level, participant-level, measurement-level, or intervention-level based on its most proximal source (Table 4).

**Table 4.** Classification of self-reported methodological limitations identified across the included studies evaluating digital interventions for illicit substance use disorders (N=56)^a^.

Domain	Description and key issues	Reference
Study-level		
Structural research design	The structural limitations arising from the lack of a control group, the absence of random assignment, and the use of a single-group design undermine the overall methodological rigor of the study.	[22,23,25,29,36,49,51-58,61-63,67,70-73,75]
Constraints on causal inference	Due to the inability to control for unmeasured confounding variables or concurrent treatment effects, this study is limited in its capacity to establish a definitive causal relationship for the intervention.	[25,30,31,34,40,41,47,48,55,57,63,68,73]
Limitations in analytical strategy	The statistical approaches used, including multiple testing, post hoc analysis, and exploratory analysis, along with the initial establishment of the research objectives, present inherent limitations to the study.	[39,57,59,75,77]
Participant-level		
Insufficient sample size	The limited sample size and insufficient statistical power restrict the feasibility of subgroup analyses and other numerical evaluations.	[23,25,27,31,33,39-41,46,48,50,51,55,59,61,64-66,71-75]
Biases in sample composition	The study is limited by the compromised representativeness of the sample resulting from recruitment bias, voluntary participation, and demographic imbalances.	[25-27,29,30,32,36,37,39,40,46,47,49,51,53,54,56,58,60-62,64,65,71,72,74,77]
Contextual generalizability constraints	The special conditions affecting external validity, such as high attrition rates, the COVID-19 pandemic, and specific legal environments, limit the generalizability of the findings.	[22,24,28,29,37,38,42,43,45,48-50,52,56-59,62,67-70,72,73,76,77]
Measurement-level		
Deficits in instrument validity	The qualitative limitations stemming from the reliance on self-reported data, recall bias, and the use of unvalidated scales hinder the reliability of the assessment tools.	[22,25,27,29,30,33-35,41,42,45-47,49-51,54,58,63-65,67,69-71,73,74,76,77]
Incomplete data coverage	The limitations regarding data availability, such as the failure to measure specific variables, missing outcome data, and the absence of formal diagnostic assessments, restrict the depth of the analysis.	[23,28-30,33,35,39,43,44,49,53,54,58,61,63,64,67-69,74,77]
Constraints in temporal assessment	The temporal constraints inherent in the data collection process, specifically the short follow-up duration and the absence of longitudinal outcome measures, prevent the assessment of the long-term effects of the intervention.	[22,28,30-32,42,44,46,49,55,61,62,66,67,70,72,76]
Intervention-level		
Complexity in intervention dosage	The unclear dosage of intervention and the structural limitations in isolating the independent effects of specific components within a multicomponent intervention preclude a definitive evaluation of each constituent element.	[23,25,32-34,36,37,44,51,57,61,74,75]
Challenges in participant adherence	The limitations arising from low adherence and irregular usage patterns, where participants do not engage with the intervention as intended, compromise the internal validity of the study.	[36,43,46,47,53,62,64,65,69,70,76,77]
Usability barriers	The systematic limitations hindering the standard delivery of the intervention, such as app download issues, technical glitches, and platform functional constraints, interfere with the intended implementation of the research protocol.	[25,42,51,53]

a Limitations are synthesized into study-level, participant-level, measurement-level, and intervention-level domains to inform the robust design of future clinical trials.

Study-level limitations were observed in 60.7% (34/56) of the studies, reflecting issues in research design that constrain internal validity or causal inference [22,23,25,29-31,34,36,39-41,47-49,51-59,61-63,67,68,70-73,75,77]. A substantial portion of the studies (23/56, 41.1%) acknowledged limitations associated with structural research design, including the use of observational, single-group, quasi-experimental, waitlist-controlled, or nonblinded designs that lacked an active control or comparison group [22,23,25,29,36,49,51-58,61-63,67,70-73,75]. Other studies (13/56, 23.2%) faced constraints on causal inference due to unmeasured confounders, baseline imbalances, or the inability to control for the effects of concurrent treatments [25,30,31,34,40,41,47,48,55,57,63,68,73]. A smaller subset of studies (5/56, 8.9%) reported limitations in analytical strategy, cautioning against the exploratory or post hoc nature of their analyses and the potential risks of multiple testing [39,57,59,75,77].

Participant-level limitations were frequently identified, present in 92.9% (52/56) of the included studies [22-33,36-43,45-55,57-77]. These limitations primarily constrain the external validity and generalizability of the findings. Subcategories within this domain included insufficient sample sizes (23/56, 41.1%), which often led to underpowered analyses or restricted the ability to conduct robust subgroup comparisons [23,25,27,31,33,39-41,46,48,50,51,55,59,61,64-66,71-75]. Many studies (27/56, 48.2%) also noted biases in sample composition due to reliance on self-selected or convenience samples, recruitment biases, high attrition, or demographic imbalances such as predominantly male or racially homogeneous cohorts [25-27,29,30,32,36,37,39,40,46,47,49,51,53,54,56,58,60-62,64,65,71,72,74,77]. Furthermore, contextual generalizability constraints were commonly highlighted (26/56, 46.4%), pointing out that interventions evaluated during the COVID-19 pandemic or within specific national, regional, and legal environments might not be broadly applicable to other settings [22,24,28,29,37,38,42,43,45,48-50,52,56-59,62,67-70,72,73,76,77].

Measurement-level limitations entailed substantial challenges in 76.8% (43/56) of the studies [22,23,25,27-35,39,41-47,49-51,53-55,58,61-74,76,77]. These issues centered on how outcomes or variables were assessed. Deficits in instrument validity were a major concern (29/56, 51.8%), largely driven by the predominant reliance on self-reported measures without biological verification or the use of unvalidated instruments [22,25,27,29,30,33-35,41,42,45-47,49-51,54,58,63-65,67,69-71,73,74,76,77]. Some studies (21/56, 37.5%) cited incomplete data coverage due to missing data, lack of formal diagnostic assessments, or failure to capture key outcome variables [23,28-30,33,35,39,43,44,49,53,54,58,61,63,64,67-69,74,77]. Constraints in temporal assessment were frequently reported (17/56, 30.4%), particularly the reliance on short follow-up periods or infrequent testing windows, which precluded the evaluation of sustained intervention effects or long-term abstinence [22,28,30-32,42,44,46,49,55,61,62,66,67,70,72,76].

Finally, intervention-level limitations pertained to the delivery, implementation, and usage of the digital programs, identified in 44.6% (25/56) of the studies [23,25,32-34,36,37,42-44,46,47,51,53,57,61,62,64,65,69,70,74-77]. Complexity in intervention dosage was a notable barrier (13/56, 23.2%); the widespread use of multicomponent intervention packages made it challenging to disentangle which specific active elements contributed to the observed effects, and the precise exposure dose remained unclear [23,25,32-34,36,37,44,51,57,61,74,75]. Several studies (12/56, 21.4%) reported challenges in participant adherence, emphasizing inconsistent usage patterns, low module completion rates, and rapid declines in engagement over the study period [36,43,46,47,53,62,64,65,69,70,76,77]. Additionally, a few studies (4/56, 7.1%) explicitly highlighted usability barriers, including technical issues, app compatibility problems, and difficulties in navigating the platform [25,42,51,53].

The limitations acknowledged by the authors of the included studies are mapped across the 4 target substance categories (Figure 4). Each study was assigned to a single substance category on the basis of the target substances reported in Table 1; studies targeting more than one substance are grouped under polysubstance. Red, yellow, and blue indicate high (≥70%), moderate (41%‐69%), and low (≤40%) prevalence. Participant-level limitations were the most frequently acknowledged overall, reported by every opioid study (6/6) [23,55,61,64,67,75] and every cannabis study (13/13) [22,28,29,31,42,43,45,56,60,65,69,74,76], by 91% (29/32) of polysubstance studies [24-27,32,33,36-41,46-51,53,54,57-59,62,66,68,70,71,73], and by 80% (4/5) of stimulant studies [30,52,72,77]. Measurement-level limitations were also widely acknowledged, ranging from 72% (23/32) of polysubstance studies [25,27,32-35,39,41,44,46,47,49-51,53,54,58,62,66,68,70,71,73] to 85% (11/13) of cannabis studies [22,28,29,31,42,43,45,65,69,74,76]. Study-level limitations were reported by all stimulant studies (5/5) [30,52,63,72,77] and by 83% (5/6) of opioid studies [23,55,61,67,75], but by only 31% (4/13) of cannabis studies [22,29,31,56]. Intervention-level limitations were the least frequently acknowledged in 3 of the 4 categories, from 20% (1/5) of stimulant studies [77] to 67% (4/6) of opioid studies [23,61,64,75].

**Figure 4.**
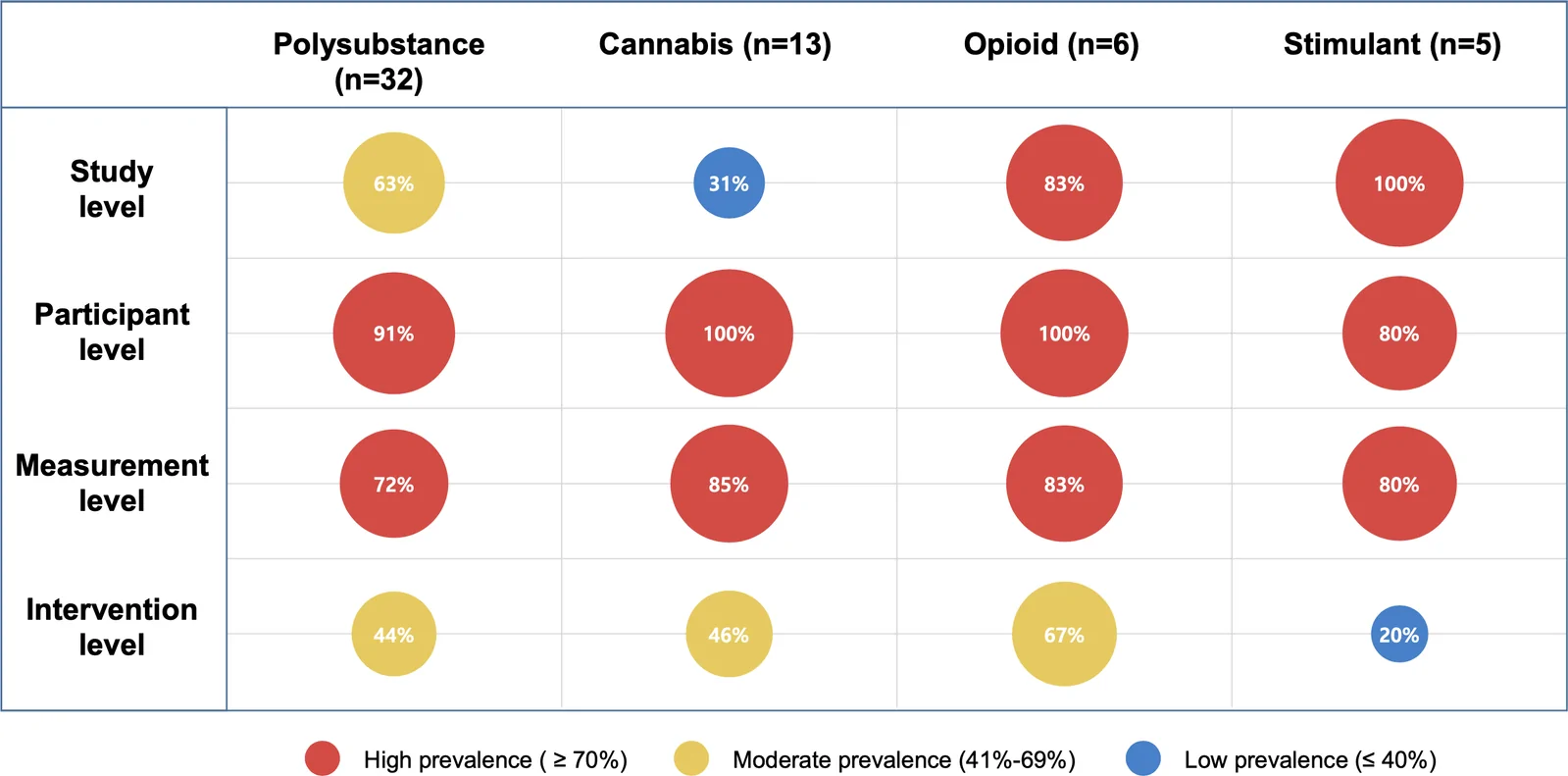
Evidence gap map showing the prevalence of methodological limitations by level and by target substance category. Bubble size, color, and internal values indicate the proportion of studies within each category (n) that acknowledged at least one limitation at the given level.

### Synthesis of Results

The limitations acknowledged across the 56 included studies clustered into 5 areas that together define where the current evidence base is least informative (Figure 5). Sampling validity corresponds to participant-level limitations; comparative positioning to study-level limitations; enhanced outcome evaluation to measurement-level limitations; and personalized content design and real-world integration to intervention-level limitations. Sampling validity was implicated by the participant-level limitations reported by 92.9% (52/56; Table 4) [22-33,36-43,45-62,64-77], which centered on small and nonrepresentative samples and on constrained generalizability. Comparative positioning followed from the study-level limitations, in particular the absence of a controlled comparison in some designs and the substantial variability in how TAU was constituted across the studies that used it. Enhanced outcome evaluation reflected the measurement-level limitations, which concerned reliance on self-reported substance use, incomplete outcome coverage, and short or absent follow-up. Personalized content design followed from the observation that generic platforms were applied across heterogeneous substance profiles, with 32 of the 56 studies enrolling polysubstance-using samples [24-27,32-41,44,46-51,53,54,57-59,62,66,68,70,71,73], along with the intervention-level limitations concerning intervention dosage. Real-world integration reflected the remaining intervention-level limitations, namely adherence and usability barriers, and the low completion rates reported in Table 2.

**Figure 5.**
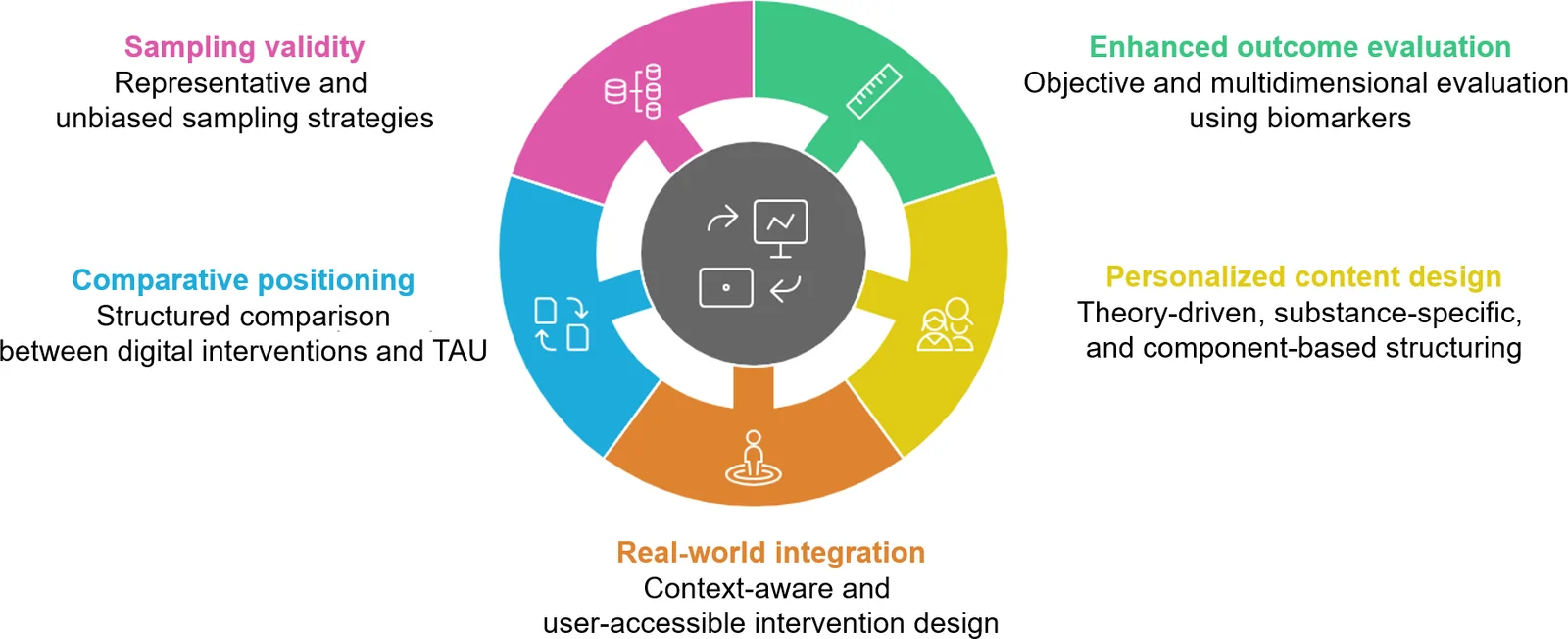
A synthesized framework summarizing the 5 areas in which the included studies most frequently acknowledged limitations, organized by the level of the limitation framework. TAU: treatment as usual.

## Discussion

### Principal Findings

This scoping review synthesized evidence from 56 published studies [22-77], drawn from 47 distinct participant samples, examining CBT-based digital interventions for illicit SUDs. Across the mutually exclusive primary target categories, polysubstance use and cannabis use were the most frequently targeted categories, accounting for 57.1% (32/56) [24-27,32-41,44,46-51,53,54,57-59,62,66,68,70,71,73] and 23.2% (13/56) of included studies [22,28,29,31,42,43,45,56,60,65,69,74,76], respectively. Opioids and stimulants were less frequently targeted, accounting for 10.7% (6/56) [23,55,61,64,67,75] and 8.9% (5/56) [30,52,63,72,77], respectively; no studies identified hallucinogen use as the primary clinical target. Despite this heterogeneity in target populations, most interventions adopted a standardized design. These mutually exclusive primary target categories should be distinguished from the polysubstance subprofiles discussed below. In this study, the patterns of concomitant use were investigated within the polysubstance studies but did not constitute additional substance-specific study categories or counts.

Because stimulant and opioid users exhibit substantially different craving dynamics, relapse risk factors, and withdrawal profiles [78], the current reliance on generic intervention structures may compromise treatment precision and potential efficacy. Furthermore, although our results identified 3 distinct clinical profiles within the polysubstance use category, most of the included studies did not tailor their interventions to these specific patterns. Instead, standard intervention structures, such as comprehensive CBT or CM packages, were applied uniformly across the different profiles. For example, studies addressing the first polysubstance subprofile—opioid and cocaine co-use, frequently observed in medication-assisted treatment settings—applied generic versions of computer-assisted therapy (Therapeutic Education System or CBT4CBT) without structural adaptation to medication adherence or opioid withdrawal dynamics [37,38,44,50]. Studies targeting the second polysubstance subprofile (cannabis-dominant use co-occurring with stimulants/cocaine) and the third polysubstance subprofile (broadly mixed illicit use) similarly relied on the same generalized digital platforms rather than profile-specific content [33,40,41,73]. Taken together, these findings indicate that the current evidence base offers little guidance on how digital interventions should be structured across polysubstance profiles. To address these limitations, digital interventions that incorporate drug-specific factors would need to be developed [79].

Combining cognitive restructuring modules with motivational enhancements or RW-based reinforcement fundamentally supports both behavioral and psychosocial outcomes by addressing multifaceted clinical needs [22,24]. Cognitive techniques effectively target distorted thought patterns and craving triggers, while motivational frameworks foster initial treatment readiness, and CM systems sustain long-term engagement [14,80,81]. However, limited transparency about how these complex theoretical models are operationalized in digital delivery formats poses a critical challenge [82]. In practice, these theoretical frameworks are often bundled into multicomponent intervention packages [23,25,32-34,36,37,44,51,61,74,75]. This widespread use of bundled packages creates a significant barrier to understanding intervention dosage, making it exceptionally difficult to disentangle which specific active components independently contribute to the observed clinical effects [83].

Clinically, 98.2% (55/56) of the reviewed programs incorporated a core TI module [22-61,63-77]. Additional engagement features such as STN and RW were present in 44.6% (25/56) [22,27,28,30,42,43,45-49,52-54,60,62-65,69-71,75-77] and 42.9% (24/56) of the interventions [23-25,32-36,39,44,51-54,60-65,70,72,74,75], respectively, while automated SF mechanisms were used in 37.5% (21/56) [22,25,27,28,30,39,42-44,46-49,53,56,65,69,71,74-76]. Although combining these multiple functional components was commonly reported to be associated with improved treatment retention and user adherence, the explicit tailoring of these features to individual risk severity or distinct substance types remains rare. Furthermore, outcome evaluations predominantly focused on reductions in substance use behavior, which served as the primary measure in 73.2% (41/56) of the studies [22-24,26-34,36-38,40,42-52,54,59,62,63,65,67,69-71,73-77]. While these results suggest that digital tools may support holistic recovery, the considerable variability in the definition of TAU across clinical trials complicates the interpretation of independent intervention effects. In clinical research, control groups frequently receive TAU to serve as a comparative baseline [84]; however, the definition of TAU fluctuates among studies. In certain trials, it encompasses highly active interventions, including face-to-face psychosocial counseling and standard pharmacotherapy [23,24,26,30-38,40,41,44,46,47,50,51,56,59,62,64,71-75], whereas in others, it consists merely of standard educational materials or waitlist conditions [22,25,27,28,39,42,43,45,48,60,65,69,76,77]. Because these comparative baselines are inconsistent, it is difficult to establish the independent clinical benefit attributable to the digital platform. A digital tool evaluated against an intensive control condition may appear less effective than a comparable tool evaluated against a minimal control condition [85]. These inconsistencies emphasize the necessity of standardized reporting and clear definitions of baseline conditions to assess the efficacy of digital therapeutics.

We categorized the methodological limitations explicitly reported in the existing literature to provide foundational insights for future trial designs. To advance the clinical utility of digital interventions, we suggest that future research address the methodological limitations related to measurement and intervention. An overreliance on self-reported measures introduces substantial risks of recall and social desirability biases [86]. Because digital interventions inherently entail lower levels of clinician control than do face-to-face formats, objective outcome metrics should be incorporated from the inception of the trial design [87]. Measurements should combine self-reports with objective indicators, including urine or saliva testing and EMA, to reduce recall bias and trace proximal mechanisms [73,86]. Furthermore, task-based behavioral response assessments could serve as robust objective indicators of effectiveness for patients with SUDs [41]. Since short-term reductions in substance use may not adequately capture broader clinical impacts, a comprehensive assessment framework featuring extended follow-up periods and multidimensional outcomes is needed to accurately predict sustained engagement and relapse prevention [88]. Since these limitations may sometimes be underreported or omitted, they should not be interpreted as an objective assessment of the study’s quality [21].

The evaluation of intervention content also requires rigorous methodological refinement. As noted, the prevalent use of multicomponent packages makes it exceptionally difficult to isolate the effects of individual components. It could be suggested that future research consider optimization-focused designs as forward-looking approaches to help disentangle these bundled features. For instance, the multiphase optimization strategy represents a conceptual framework that could allow future investigators to empirically screen multiple active ingredients [83]. Similarly, for just-in-time adaptive interventions, microrandomized trials offer a hypothetical approach to evaluate digital prompts in naturalistic settings [79,89]. Although advanced frameworks such as factorial trials within the multiphase optimization strategy or microrandomized trials extend beyond the empirical scope of the studies mapped in this review, they illustrate promising future directions for exploring the optimal dose and timing of digital therapeutics. Empirical examination of dose-response relationships between intervention intensity and user engagement is highly warranted [36]. From a clinical-design perspective, future studies may benefit from documenting TAU components, intensity, and integration strategies in sufficient detail. Using specific reporting formats such as the TIDieR (Template for Intervention Description and Replication) guidelines can facilitate the precise evaluation of combined effects [90]. Comparative designs that clearly distinguish digital-only, standard care, and combined approaches will clarify relative effectiveness, while hybrid effectiveness-implementation trials can incorporate cost and scalability questions relevant to decision makers [90,91]. Finally, structural tailoring must account for drug-specific differences in pharmacodynamics, usage motivations, and contextual risk factors through explicit theory-to-feature mapping [79].

### Future Directions and Author Recommendations

We identify several implementation considerations that may merit attention in future research on the clinical applicability of digital interventions. These considerations could include technological accessibility, interoperability with health information systems, and clear data governance. Future studies might explore how digital interventions could be embedded within stepped-care pathways and existing clinical workflows, and whether such integration may support their adoption and sustainment. Although clinician-facing dashboards were described as platform features in some reviewed studies, including reSET and CHAMPS (Cannabis Harm-reducing App to Manage Practices Safely) [24,36,44,56,61], this review did not systematically evaluate their implementation or clinical utility. Beyond the features mapped in this review, automated escalation protocols [92], interoperability with electronic health records and pharmacy systems [93], privacy safeguards, and transparent consent procedures may be considered as forward-looking implementation options. These proposals are intended to guide future research and should not be interpreted as evidence-based conclusions of this review.

### Limitations

Several methodological limitations of this scoping review should be noted. First, the literature search was restricted to peer-reviewed journal articles and excluded gray literature, including conference proceedings, dissertations, and unpublished institutional reports. This criterion was applied to ensure the methodological rigor and quality of the included evidence. However, in the rapidly developing field of digital therapeutics, this exclusion may introduce a risk of publication bias. As a result, the interventions identified in this review may not fully represent the range of digital interventions currently being developed or implemented, and some recent or practice-based programs may have been omitted. To partly mitigate this limitation, we broadened our search to 6 bibliographic databases and conducted citation searching; nonetheless, this possibility should be considered when interpreting the scope and representativeness of the identified interventions. Second, consistent with the standard methodology of scoping reviews, no formal critical appraisal of the included studies was conducted. Consequently, the intervention outcomes summarized herein reflect the reported findings at a purely descriptive level. Conclusions regarding the definitive reliability or comparative clinical significance of these outcomes must therefore be interpreted with appropriate caution. Finally, this review primarily focused on mapping the structural and theoretical dimensions of digital interventions and did not systematically examine user-level contextual factors. Variables such as gender, age, baseline disorder severity, and digital accessibility or receptivity can significantly influence treatment outcomes and user adherence. Future systematic reviews and clinical trials should adopt more granular reporting standards and conduct stratified analyses to better account for these critical moderating variables.

### Conclusion

This scoping review synthesized evidence from 56 studies evaluating digital interventions based on CBT for illicit SUDs [22-77]. Unlike previous reviews, which have largely been confined to a single substance or to a single delivery platform, this review is innovative in mapping interventions across the full spectrum of illicit substances—including polysubstance use, which reflects the clinical reality of most addiction services—and in linking the functional components of each intervention to their intended therapeutic mechanisms rather than classifying them by delivery format alone. Almost all included studies incorporated a core TI, and 2 or more of the functional components were combined and incorporated into the digital interventions. However, current results have shown limitations in clearly distinguishing the clinical efficacy of digital interventions due to the widespread application of general interventions, an excessive dependence on self-report measurement, and inconsistencies in TAU conditions. Additionally, combining multiple functional components obscured the core components involved in treatment, making it difficult to identify specific mechanisms of clinical intervention.

Therefore, this scoping review contributes to the development of digital intervention technologies for illicit SUDs by describing and comparing CBT-based digital interventions across a range of substances of abuse. Furthermore, by synthesizing the limitations reported by the authors of the included studies, it proposes methodological improvements for the future evaluation of these technologies. For these interventions to become a scalable and effective treatment modality in real-world clinical settings, future studies must emphasize substance-specific customization, obtain objective outcome measures, and adopt modular trial designs. Taken together, these findings indicate that CBT-based digital interventions are, at present, best positioned as adjuncts that extend the reach and continuity of existing addiction care rather than as stand-alone substitutes for it. By addressing these methodological limitations, digital therapeutics may develop into a sustainable, patient-oriented treatment technology in the field of addiction treatment.

## Supplementary material

10.2196/99718 Multimedia Appendix 1 Charting form.

10.2196/99718 Multimedia Appendix 2 Search strategies reported in accordance with the PRISMA-S extension (Rethlefsen et al, 2021). This appendix presents the complete, line-by-line search strategy executed in each database, together with the number of records retrieved at every step.

10.2196/99718 Multimedia Appendix 3 Operationalization and heterogeneity of treatment-as-usual across included studies (n=38).

10.2196/99718 Checklist 1 PRISMA-ScR checklist.

## Abbreviations

CBT: cognitive behavioral therapy
CBT4CBT: Computer-Based Training for Cognitive Behavioral Therapy
CHAMPS: Cannabis Harm-reducing App to Manage Practices Safely
CM: contingency management
CRA: community reinforcement approach
DBT: dialectical behavior therapy
*DSM-5*: *Diagnostic and Statistical Manual of Mental Disorders* (*Fifth Edition*)
EMA: ecological momentary assessment
*ICD-10*: *International Statistical Classification of Diseases, Tenth Revision*
*ICD-11*: *International Statistical Classification of Diseases, 11th Revision*
IVR: interactive voice response
JBI: Joanna Briggs Institute
MBT: mindfulness-based therapy
MI: motivational interviewing
PRISMA-ScR: Preferred Reporting Items for Systematic Reviews and Meta-Analyses extension for Scoping Reviews
RCT: randomized controlled trial
RW: reward
SF: support and feedback
STN: self-tracking and notification
SUD: substance use disorder
T-CBT: traditional cognitive behavioral therapy
TAU: treatment as usual
TI: therapeutic intervention
TIDieR: Template for Intervention Description and Replication
