# Supplementary material for: Cognitive Behavioral Therapy–Based Digital Interventions for Illicit Substance Use Disorders: Scoping Review of Intervention Design, Efficacy Indicators, and Methodological Gaps
Source: J Med Internet Res. 2026 Nov 3;28:e99718. doi: 10.2196/99718 (PMC13730295; doi:10.2196/99718)
Supplement: Supplementary file 1 [file jmir-v28-e99718-s002.pdf]

Multimedia Appendix 2. Search strategies

Reported in accordance with the PRISMA-S extension (Rethlefsen et al., 2021). This appendix presents the complete, line-by-line search strategy executed in each database, together with the number of records retrieved at every step.

Search reporting summary

**Databases and platforms searched**	PubMed/MEDLINE (National Library of Medicine); Embase (Elsevier); APA PsycINFO (ProQuest); Web of Science Core Collection (Clarivate); Cochrane Library (Wiley); CINAHL (EBSCO).
**Coverage searched**	Each database was searched from its inception to 28 June 2026.
**Date searches executed**	28 June 2026. All six database searches were re-executed in full on this date using the final strategy.
**Limits and filters applied**	None. No language, date, publication-type, or study-design limits or filters were applied to any database search. Eligibility restrictions were applied at the screening stage only.
**Controlled vocabulary used**	MeSH (PubMed, Cochrane Library); Emtree (Embase); APA Thesaurus of Psychological Index Terms (PsycINFO); CINAHL Subject Headings (CINAHL). The Web of Science Core Collection has no controlled vocabulary, and the strategy for that database therefore used free-text terms alone.
**Structure of the strategy**	Three concepts — substance use disorder, cognitive behavioural therapy, and digital health — were each represented by controlled-vocabulary terms combined with a broad set of free-text synonyms. Terms were combined with OR within each concept and with AND across concepts. Truncation and the field modifiers, phrase operators, and proximity operators appropriate to each platform were applied.
**Strategy development and execution**	The search strategy was developed by JWC, SMK, SJJ, and MJK. The searches in all six databases were executed by SMK, SJJ, and MJK, who also undertook the screening of the retrieved records.
**Peer review of the strategy**	Before the final searches were run, the strategy was peer reviewed against the Peer Review of Electronic Search Strategies (PRESS) checklist by YHC, DJK, NRL, and KH, none of whom had been involved in developing or executing the strategy. Their comments addressed the coverage of the substance-use terminology and the scope of the digital health concept.
**Supplementary search methods**	Backward and forward citation searching was undertaken on 3 July 2026; the methods and yield are reported in the Methods and Results and in Figure 1.
**Sources not searched**	No trial registers were searched and no sources of gray literature were sought.

Records retrieved by database

Database	Platform / vendor	Coverage searched	Date searched	Records retrieved
PubMed/MEDLINE	National Library of Medicine	Inception to 28 June 2026	28 June 2026	224
Embase	Elsevier	Inception to 28 June 2026	28 June 2026	674
Web of Science Core Collection	Clarivate	Inception to 28 June 2026	28 June 2026	153
APA PsycINFO	ProQuest	Inception to 28 June 2026	28 June 2026	237
Cochrane Library	Wiley	Inception to 28 June 2026	28 June 2026	224
CINAHL	EBSCO	Inception to 28 June 2026	28 June 2026	164
**Total**				**1,676**

Records retrieved before deduplication. Deduplication and screening are reported in the Results and in Figure 1.

1. PubMed/MEDLINE

Platform: National Library of Medicine | Coverage searched: inception to 28 June 2026 | Date of search: 28 June 2026 | Records retrieved: 224

Search #	Exact query	Results
#1	"Substance-Related Disorders"[MeSH Terms]	336,258
#2	"substance use disorder*"[Title/Abstract] OR "drug use disorder*"[Title/Abstract] OR "drug dependenc*"[Title/Abstract] OR "drug addict*"[Title/Abstract] OR "drug misuse*"[Title/Abstract] OR "drug abuse*"[Title/Abstract] OR "opioid use disorder*"[Title/Abstract] OR "cocaine use disorder*"[Title/Abstract] OR "amphetamine use disorder*"[Title/Abstract] OR "hallucinogen use disorder*"[Title/Abstract] OR "cannabis use disorder*"[Title/Abstract] OR "illicit drug use*"[Title/Abstract]	130,395

Search #	Exact query	Results
	OR "narcotic abuse*"[Title/Abstract] OR "prescription drug misuse*"[Title/Abstract] OR "opioid use*"[Title/Abstract] OR "heroin use*"[Title/Abstract] OR "fentanyl use*"[Title/Abstract] OR "morphine use*"[Title/Abstract] OR "oxycodone use*"[Title/Abstract] OR "methadone use*"[Title/Abstract] OR "cocaine use*"[Title/Abstract] OR "amphetamine use*"[Title/Abstract] OR "methamphetamine use*"[Title/Abstract] OR "mdma use*"[Title/Abstract] OR "hallucinogen use*"[Title/Abstract] OR "lsd use*"[Title/Abstract] OR "psilocybin* use"[Title/Abstract] OR "ketamine* use"[Title/Abstract] OR "pcp use*"[Title/Abstract] OR "ecstasy use*"[Title/Abstract] OR "cannabis use*"[Title/Abstract] OR "marijuana use*"[Title/Abstract] OR "cannabinoid use*"[Title/Abstract]	
#3	#1 OR #2	393,584
#4	"Cognitive Behavioral Therapy"[MeSH Terms]	44,296
#5	"cognitive behavioral therap*"[Title/Abstract] OR "CBT"[Title/Abstract] OR "cognitive behavior therap*"[Title/Abstract] OR "cognitive therap*"[Title/Abstract] OR "behavioral therapy cognitive"[Title/Abstract] OR "behavioral intervention*"[Title/Abstract] OR "behavior therap*"[Title/Abstract] OR "acceptance and commitment therap*"[Title/Abstract] OR "mindfulness based cognitive therap*"[Title/Abstract] OR "cognitive restructuring*"[Title/Abstract]	52,801
#6	#4 OR #5	76,507
#7	"Digital Health"[MeSH Terms]	3,837
#8	"telehealth*"[Title/Abstract] OR "ehealth*"[Title/Abstract] OR "mobile health*"[Title/Abstract] OR "digital health*"[Title/Abstract] OR "digital therapeutic*"[Title/Abstract] OR "mhealth*"[Title/Abstract] OR "virtual medicine*"[Title/Abstract] OR "digital intervention*"[Title/Abstract] OR "app based therap*"[Title/Abstract] OR "internet delivered therap*"[Title/Abstract] OR "technology assisted therap*"[Title/Abstract] OR "health technolog*"[Title/Abstract] OR "online therap*"[Title/Abstract] OR "web based therap*"[Title/Abstract] OR "DTx"[Title/Abstract]	168,868
#9	#7 OR #8	168,868
**#10**	**#3 AND #6 AND #9**	**224**

2. Embase

Platform: Elsevier | Coverage searched: inception to 28 June 2026 | Date of search: 28 June 2026 | Records retrieved: 674

Search #	Exact query	Results
#1	'drug dependence'/exp	345,424
#2	'substance use disorder*' OR 'drug use disorder*' OR 'drug dependenc*' OR 'drug addict*' OR 'drug misuse*' OR 'drug abuse*' OR 'opioid use disorder*' OR 'cocaine use disorder*' OR 'amphetamine use disorder*' OR 'hallucinogen use disorder*' OR 'cannabis use disorder*' OR 'illicit drug use*' OR 'narcotic abuse*' OR 'prescription drug misuse*' OR 'opioid use*' OR 'heroin use*' OR 'fentanyl use*' OR 'morphine use*' OR 'oxycodone use*' OR 'methadone use*' OR 'cocaine use*' OR 'amphetamine use*' OR 'methamphetamine use*' OR 'mdma use*' OR 'hallucinogen use*' OR 'lsd use*' OR 'psilocybin* use' OR 'ketamine* use' OR 'pcp use*' OR 'ecstasy use*' OR 'cannabis use*' OR 'marijuana use*' OR 'cannabinoid use*'	313,420
#3	#1 OR #2	503,697
#4	'cognitive behavioral therapy'/exp	51,528
#5	'cognitive behavioral therap*' OR 'cbt' OR 'cognitive behavior therap*' OR 'cognitive therap*' OR 'behavioral therapy, cognitive' OR 'behavioral intervention*' OR 'behavior therap*' OR 'acceptance and commitment therap*' OR 'mindfulness-based cognitive therap*' OR 'cognitive restructuring*'	185,489
#6	#4 OR #5	188,132
#7	'digital health'/exp	12,006
#8	'telehealth*' OR 'ehealth*' OR 'mobile health*' OR 'digital health*' OR 'digital therapeutic*' OR 'mhealth*' OR 'virtual medicine*' OR 'digital intervention*' OR 'app-based therap*' OR 'internet-delivered therap*' OR 'technology-assisted therap*' OR 'health technolog*' OR 'online therap*' OR 'web-based therap*' OR 'dtx'	203,790
#9	#7 OR #8	203,790
**#10**	**#3 AND #6 AND #9**	**674**

3. Web of Science Core Collection

Platform: Clarivate | Coverage searched: inception to 28 June 2026 | Date of search: 28 June 2026 | Records retrieved: 153

Search #	Exact query	Results
#1	ALL=("substance use disorder*" OR "drug use disorder*" OR "drug dependenc*" OR "drug addict*" OR "drug misuse*" OR "drug abuse*" OR "opioid use disorder*" OR "cocaine use disorder*" OR "amphetamine use disorder*" OR "hallucinogen use disorder*" OR "cannabis use disorder*" OR "illicit	219,341

Search #	Exact query	Results
	drug use*" OR "narcotic abuse*" OR "prescription drug misuse*" OR "opioid use*" OR "heroin use*" OR "fentanyl use*" OR "morphine use*" OR "oxycodone use*" OR "methadone use*" OR "cocaine use*" OR "amphetamine use*" OR "methamphetamine use*" OR "MDMA use*" OR "hallucinogen use*" OR "LSD use*" OR "psilocybin* use" OR "ketamine* use" OR "PCP use*" OR "ecstasy use*" OR "cannabis use*" OR "marijuana use*" OR "cannabinoid use*")	
#2	ALL=("cognitive behavioral therap*" OR "CBT" OR "cognitive behavior therap*" OR "cognitive therap*" OR "behavioral therapy, cognitive" OR "Behavioral Intervention*" OR "Behavior Therap*" OR "Acceptance and Commitment Therap*" OR "Mindfulness-Based Cognitive Therap*" OR "Cognitive Restructuring*")	55,316
#3	ALL=("Telehealth*" OR "eHealth*" OR "Mobile Health*" OR "Digital Health*" OR "Digital Therapeutic*" OR "mHealth*" OR "Virtual Medicine*" OR "Digital Intervention*" OR "App-based Therap*" OR "Internet-delivered Therap*" OR "Technology-assisted Therap*" OR "Health Technolog*" OR "Online Therap*" OR "Web-based Therap*" OR "DTx")	144,099
**#4**	**#1 AND #2 AND #3**	**153**

4. APA PsycINFO

Platform: ProQuest | Coverage searched: inception to 28 June 2026 | Date of search: 28 June 2026 | Records retrieved: 237

Search #	Exact query	Results
S1	MAINSUBJECT.EXACT.EXPLODE("Substance Use Disorder") OR ("substance use disorder*" OR "drug use disorder*" OR "drug dependenc*" OR "drug addict*" OR "drug misuse*" OR "drug abuse*" OR "opioid use disorder*" OR "cocaine use disorder*" OR "amphetamine use disorder*" OR "hallucinogen use disorder*" OR "cannabis use disorder*" OR "illicit drug use*" OR "narcotic abuse*" OR "prescription drug misuse*" OR "opioid use*" OR "heroin use*" OR "fentanyl use*" OR "morphine use*" OR "oxycodone use*" OR "methadone use*" OR "cocaine use*" OR "amphetamine use*" OR "methamphetamine use*" OR "MDMA use*" OR "hallucinogen use*" OR "LSD use*" OR "psilocybin* use" OR "ketamine* use" OR "PCP use*" OR "ecstasy use*" OR "cannabis use*" OR "marijuana use*" OR "cannabinoid use*")	187,923
S2	MAINSUBJECT.EXACT.EXPLODE("Cognitive Behavior Therapy") OR ("cognitive behavioral therap*" OR "CBT" OR "cognitive behavior therap*" OR "cognitive therap*" OR "behavioral therapy, cognitive" OR "Behavioral Intervention*" OR "Behavior Therap*" OR "Acceptance and Commitment Therap*" OR "Mindfulness-Based Cognitive Therap*" OR "Cognitive Restructuring*")	141,769
S3	MAINSUBJECT.EXACT.EXPLODE("Digital Health Resources") OR ("Telehealth*" OR "eHealth*" OR "Mobile Health*" OR "Digital Health*" OR "Digital Therapeutic*" OR "mHealth*" OR "Virtual Medicine*" OR "Digital Intervention*" OR "App-based Therap*" OR "Internet-delivered Therap*" OR "Technology-assisted Therap*" OR "Health Technolog*" OR "Online Therap*" OR "Web-based Therap*" OR "DTx")	31,043
**S4**	**[S1] AND [S2] AND [S3]**	**237**

5. Cochrane Library

Platform: Wiley | Coverage searched: inception to 28 June 2026 | Date of search: 28 June 2026 | Records retrieved: 224

Search #	Exact query	Results
#1	[mh "Substance-Related Disorders"]	22,317
#2	(substance NEXT use NEXT disorder*) OR (drug NEXT use NEXT disorder*) OR (drug NEXT dependenc*) OR (drug NEXT addict*) OR (drug NEXT misuse*) OR (drug NEXT abuse*) OR (opioid NEXT use NEXT disorder*) OR (cocaine NEXT use NEXT disorder*) OR (amphetamine NEXT use NEXT disorder*) OR (hallucinogen NEXT use NEXT disorder*) OR (Cannabis NEXT use NEXT disorder*) OR (illicit NEXT drug NEXT use*) OR (narcotic NEXT abuse*) OR (prescription NEXT drug NEXT misuse*) OR (opioid NEXT use*) OR (heroin NEXT use*) OR (fentanyl NEXT use*) OR (morphine NEXT use*) OR (oxycodone NEXT use*) OR (methadone NEXT use*) OR (cocaine NEXT use*) OR (amphetamine NEXT use*) OR (methamphetamine NEXT use*) OR (MDMA NEXT use*) OR (hallucinogen NEXT use*) OR (LSD NEXT use*) OR (psilocybin* NEXT use) OR (ketamine* NEXT use) OR (PCP NEXT use*) OR (ecstasy NEXT use*) OR (cannabis NEXT use*) OR (marijuana NEXT use*) OR (cannabinoid NEXT use*)	21,157
#3	#1 OR #2	36,613
#4	[mh "Cognitive Behavioral Therapy"]	15,990
#5	(cognitive NEXT behavioral NEXT therap*) OR CBT OR (cognitive NEXT behavior NEXT therap*) OR (cognitive NEXT therap*) OR behavioral therapy, cognitive OR (Behavioral NEXT Intervention*) OR (Behavior NEXT Therap*) OR "Acceptance and Commitment Therapy" OR (Mindfulness-Based NEXT Cognitive NEXT Therap*) OR (Cognitive NEXT Restructuring*)	55,378
#6	#4 OR #5	57,466
#7	[mh "Digital Health"]	129
#8	Telehealth* OR eHealth* OR (Mobile NEXT Health*) OR (Digital NEXT Health*) OR (Digital NEXT Therapeutic*) OR mHealth* OR (Virtual NEXT Medicine*) OR (Digital NEXT Intervention*) OR (App-based NEXT Therap*) OR (Internet-delivered NEXT Therap*) OR (Technology-assisted NEXT Therap*)	20,812

Search #	Exact query	Results
	OR (Health NEXT Technolog*) OR (Online NEXT Therap*) OR (Web-based NEXT Therap*) OR DTx	
#9	#7 OR #8	20,812
**#10**	**#3 AND #6 AND #9**	**224**

6. CINAHL

Platform: EBSCO | Coverage searched: inception to 28 June 2026 | Date of search: 28 June 2026 | Records retrieved: 164

Search #	Exact query	Results
S1	MH "Substance Use Disorders+"	203,855
S2	"substance use disorder*" OR "drug use disorder*" OR "drug dependenc*" OR "drug addict*" OR "drug misuse*" OR "drug abuse*" OR "opioid use disorder*" OR "cocaine use disorder*" OR "amphetamine use disorder*" OR "hallucinogen use disorder*" OR "Cannabis use disorder*" OR "illicit drug use*" OR "narcotic abuse*" OR "prescription drug misuse*" OR "opioid use*" OR "heroin use*" OR "fentanyl use*" OR "morphine use*" OR "oxycodone use*" OR "methadone use*" OR "cocaine use*" OR "amphetamine use*" OR "methamphetamine use*" OR "MDMA use*" OR "hallucinogen use*" OR "LSD use*" OR "psilocybin* use" OR "ketamine* use" OR "PCP use*" OR "ecstasy use*" OR "cannabis use*" OR "marijuana use*" OR "cannabinoid use*"	105,300
S3	S1 OR S2	227,341
S4	MH "Cognitive Therapy+"	37,063
S5	"cognitive behavioral therap*" OR "CBT" OR "cognitive behavior therap*" OR "cognitive therap*" OR "behavioral therapy, cognitive" OR "Behavioral Intervention*" OR "Behavior Therap*" OR "Acceptance and Commitment Therap*" OR "Mindfulness-Based Cognitive Therap*" OR "Cognitive Restructuring*"	50,547
S6	S4 OR S5	61,351
S7	MH "Digital Health+"	44,426
S8	"Telehealth*" OR "eHealth*" OR "Mobile Health*" OR "Digital Health*" OR "Digital Therapeutic*" OR "mHealth*" OR "Virtual Medicine*" OR "Digital Intervention*" OR "App-based Therap*" OR "Internet-delivered Therap*" OR "Technology-assisted Therap*" OR "Health Technolog*" OR "Online Therap*" OR "Web-based Therap*" OR "DTx"	58,434
S9	S7 OR S8	77,789
**S10**	**S3 AND S6 AND S9**	**164**
