# Supplementary material for: Cognitive Behavioral Therapy–Based Digital Interventions for Illicit Substance Use Disorders: Scoping Review of Intervention Design, Efficacy Indicators, and Methodological Gaps
Source: J Med Internet Res. 2026 Nov 3;28:e99718. doi: 10.2196/99718 (PMC13730295; doi:10.2196/99718)
Supplement: Supplementary file 3 [file jmir-v28-e99718-s003.docx]

Multimedia Appendix 3**.** Operationalization and Heterogeneity of Treatment-as-Usual (TAU) Across Included Studies (n=38)

Study	Clinical Setting & Target	TAU Operationalization & Key Components	Frequency / Intensity (Dose)

1. **Medication-Assisted Treatment + Counseling**

Bickel et al. [44]	Outpatient (Opioid, Cocaine)	Buprenorphine/naloxone + standard counseling.	Standard therapy sessions.

Marsch et al. [37], Guarino et al. [23], Kim et al. [38]

Methadone Maintenance Treatment (Opioid)	Daily methadone (80-100 mg/day) + individual counseling with

certified counselors (CASACs).

30-60 mins weekly for 1st month, then bi-weekly.

Moore et al. [47]	OTP Clinic (Opioid)	Daily methadone + open access therapeutic groups + optional services (psychiatric/medical care).

Min. 1 group session/month.

Garland et al. [55]	Outpatient (Opioid)	Buprenorphine or Methadone + standard clinical management.	Standard clinic schedules.

Stidham et al. [61]	Outpatient (Opioid)	MOUD (Medications for Opioid Use Disorder) through usual outpatient care.

Kawasaki et al. [64]	Opioid Treatment Program (Opioid)	Methadone or Buprenorphine + monthly medical visits +

telehealth/in-person counseling.

MOUD dispensing schedules.

≥2.5 hours/month counseling.

2. **Intensive Outpatient Programs & Structured Psychotherapy**

Lévesque et al. [34]	Outpatient Addiction Treatment (SUD)	Standard counselor-delivered individual and group therapy.	4 to 12 hours/week.

DeFulio et al. [75]	Outpatient Treatment Center (Opioid)	IOP, OP, or CC step-down care + buprenorphine/naltrexone +

individual/group counseling.

Maricich et al. [24]	Outpatient Treatment (Polysubstance)	Intensive outpatient treatment consisting of individual and group

face-to-face counseling.

Paquette et al. [26]	Intensive Outpatient Program (SUD)	Abstinence-based IOP based on the Matrix Model (Group therapy

+ weekly case management).

Patel et al. [59]	Mental Health Hospital (Polysubstance)	Group therapy (primarily CBT, with MI and DBT concepts) +

individual therapy as needed.

Varies: 3 groups/week down to 1 group/month. Average 4-6 hours/week (min. 2 hours/week).

3 hours/day, 3 days/week for 12 weeks.

8 to 12 sessions total.

3. **General Outpatient & Residential Counseling**

Carroll et al. [73]	Community Clinic (Polysubstance)	General drug counseling (individual and group) + urine/breath

specimens.

Weekly individual and group sessions.

Campbell et al. [32,33], Marino et al. [35], Luderer et al. [36]

Outpatient Addiction Treatment (SUD)	General outpatient counseling and treatment groups.	Average 1.5 to 2 sessions/week.

Budney et al. [74]	Outpatient (Cannabis)	Brief Motivational Enhancement Therapy (MET) only condition.	Two 60-75 min sessions total.

Tiburcio et al. [46]	Treatment Center (Polysubstance)	Face-to-face CBT interventions ordinarily offered at the

participating center.

8 weekly sessions.

Liang et al. [71], Zhang et al. [72]

Community-based Rehab (SUD)	Telephone counseling from social workers covering work, family,

and stress management.

Weekly sessions (average 11 mins/call).

Kelpin et al. [50]	Residential Treatment (Polysubstance)	Standard residential treatment protocol for SUDs.	Daily residential programming. Chun-Hung et al. [30]	Psychiatric Addiction Unit (SUD)	Standard treatment provided by the psychiatric facility.	Standard facility schedules.

Elison et al. [66], Elison- Various Outpatient / Publicly Funded Settings (SUD)	Routine outpatient standard care based on individual facility	Varies by individual treatment facility protocols.

Davies et al. [29,67], Günther et al. [68], Downie et al. [57], Zhang & Hardcastle [62], Cardoso Sao Mateus et al. [63]

4. **Specialized or Culturally Tailored Care**

protocols without specific digital enhancements.

Paris et al. [40], Benitez et al. [41]

Outpatient Services for Latinos / Spanish-speaking	Supportive counseling delivered via weekly group or individual

sessions tailored for Latino patients.

Weekly group/individual sessions.

Campbell et al. [51]	Urban Indian Health Care (SUD)	Matrix model IOP incorporating AI/AN cultural activities (e.g.,

Red Road to Sobriety, drumming).

Coronado-Montoya et al. [56]	Early Intervention Services (Cannabis)	Standard EIS for First-Episode Psychosis (FEP), including

psychosocial and pharmacological care.

6 groups/week + individual sessions. Synchronized with EIS appointments.

5. **Real-World Observational & Minimal Care**

Baumgartner et al. [42]	Web-based (Cannabis)	'Internet as usual' condition providing standard online educational

resources.

Wilde et al. [53]	Community / Justice System (SUD)	Real-world recovery support services for justice-referred and self-

referred participants.

Xiong et al. [54]	Real-world outpatient (SUD)	Unconstrained real-world outpatient clinical care under

prescribing clinicians.

Self-paced / minimal.

Highly variable (self-directed).

Varies by clinician and patient needs.

Abbreviations: AI/AN = American Indian/Alaska Native; CASAC = Certified Alcohol and Substance Abuse Counselor; CBT = Cognitive Behavioral Therapy; CC = Continuing Care; DBT = Dialectical Behavior Therapy; EIS = Early Intervention Services; IOP = Intensive Outpatient Program; MAT/MOUD = Medication-Assisted Treatment / Medications for Opioid Use Disorder; MET = Motivational Enhancement Therapy; MI = Motivational Interviewing; OP = Outpatient Program; OTP = Opioid Treatment Program; SUD = Substance Use Disorder

**23.** Guarino H, Acosta M, Marsch LA, Xie H, Aponte-Melendez Y. A mixed-methods evaluation of the feasibility, acceptability, and preliminary efficacy of a mobile intervention for methadone maintenance clients. Psychol Addict Behav. Feb 2016;30(1):1-11. [doi: 10.1037/adb0000128] [Medline: 26618796]

**24.** Maricich YA, Nunes EV, Campbell ANC, Botbyl JD, Luderer HF. Safety and efficacy of a digital therapeutic for substance use disorder: Secondary analysis of data from a NIDA clinical trials network study. Subst Abus. 2022;43(1):937-942. [doi: 10.1080/08897077.2022.2060425] [Medline: 35420979]

**26.** Paquette CE, Reese ED, Yi JY, Maccarone JM, Stewart ZJ, Daughters SB. Group behavioral activation with and without a smartphone app in intensive outpatient treatment for substance use disorder: A three-arm randomized controlled trial. Drug Alcohol Depend. Feb 1 2023;243:109758. [doi: 10.1016/j.drugalcdep.2022.109758] [Medline: 36634574]

**29.** Elison-Davies S, Wardell JD, Quilty LC, Ward J, Davies G. Examining correlates of cannabis users' engagement with a digital intervention for substance use disorder: An observational study of clients in UK services delivering Breaking Free Online. J Subst Abuse Treat. Apr 2021;123:108261. [doi: 10.1016/j.jsat.2020.108261] [Medline: 33612195]

**30.** Chun-Hung L, Guan-Hsiung L, Wu-Chuan Y, Yu-Hsin L. Chatbot-assisted therapy for patients with methamphetamine use disorder: a preliminary randomized controlled trial. Front Psychiatry. 2023;14:1159399. [doi: 10.3389/fpsyt.2023.1159399] [Medline: 37484677]

**32.** Campbell AN, Nunes EV, Matthews AG, Stitzer M, Miele GM, Polsky D, et al. Internet-delivered treatment for substance abuse: a multisite randomized controlled trial. Am J Psychiatry. Jun 2014;171(6):683-690. [doi: 10.1176/appi.ajp.2014.13081055] [Medline: 24700332]

**33.** Campbell AN, Nunes EV, Pavlicova M, Hatch-Maillette M, Hu MC, Bailey GL, et al. Gender-based Outcomes and Acceptability of a Computer-assisted Psychosocial Intervention for Substance Use Disorders. J Subst Abuse Treat. Jun 2015;53:9-15. [doi: 10.1016/j.jsat.2014.12.006] [Medline: 25613105]

**34.** Levesque A, Campbell AN, Pavlicova M, Hu MC, Walker R, McClure EA, et al. Coping strategies as a mediator of internet-delivered psychosocial treatment: Secondary analysis from a NIDA CTN multisite effectiveness trial. Addict Behav. Feb 2017;65:74-80. [doi: 10.1016/j.addbeh.2016.09.012] [Medline: 27776269]

**35.** Marino LA, Campbell ANC, Pavlicova M, Hu M, Nunes EV. Social functioning outcomes among individuals with substance use disorders receiving internet-delivered community reinforcement approach. Subst Use Misuse. 2019;54(7):1067-1074. [doi: 10.1080/10826084.2018.1528458] [Medline: 30849925]

**36.** Luderer HF, Campbell ANC, Nunes EV, Enman NM, Xiong X, Gerwien R, et al. Engagement patterns with a digital therapeutic for substance use disorders: Correlations

with abstinence outcomes. J Subst Abuse Treat. Jan 2022;132:108585. [doi: 10.1016/j.jsat.2021.108585] [Medline: 34366201]

**37.** Marsch LA, Guarino H, Acosta M, Aponte-Melendez Y, Cleland C, Grabinski M, et al. Web-based behavioral treatment for substance use disorders as a partial replacement of standard methadone maintenance treatment. J Subst Abuse Treat. Jan 2014;46(1):43-51. [doi: 10.1016/j.jsat.2013.08.012] [Medline: 24060350]

**38.** Kim SJ, Marsch LA, Acosta MC, Guarino H, Aponte-Melendez Y. Can persons with a history of multiple addiction treatment episodes benefit from technology delivered behavior therapy? A moderating role of treatment history at baseline. Addict Behav. Mar 2016;54:18-23. [doi: 10.1016/j.addbeh.2015.11.009] [Medline: 26657820]

**40.** Paris M, Silva M, Anez-Nava L, Jaramillo Y, Kiluk BD, Gordon MA, et al. Culturally Adapted, Web-Based Cognitive Behavioral Therapy for Spanish-Speaking Individuals With Substance Use Disorders: A Randomized Clinical Trial. Am J Public Health. Nov 2018;108(11):1535-1542. [doi: 10.2105/AJPH.2018.304571] [Medline: 30252519]

**41.** Benitez B, Loya JM, Jaramillo Y, Muro-Rodriguez NJ, Rojas Perez OF, Nich C, et al. Improvement in coping skills from culturally-adapted digital CBT for Spanish-speaking Hispanics with substance use disorder: Secondary analysis of a randomized clinical trial. J Subst Use Addict Treat. Jan 2025;168:209536. [doi: 10.1016/j.josat.2024.209536] [Medline: 39374899]

**42.** Baumgartner C, Schaub MP, Wenger A, Malischnig D, Augsburger M, Walter M, et al. CANreduce 2.0 Adherence-Focused Guidance for Internet Self-Help Among Cannabis Users: Three-Arm Randomized Controlled Trial. J Med Internet Res. Apr 30 2021;23(4):e27463. [doi: 10.2196/27463] [Medline: 33929333]

**44.** Bickel WK, Marsch LA, Buchhalter AR, Badger GJ. Computerized behavior therapy for opioid-dependent outpatients: a randomized controlled trial. Exp Clin Psychopharmacol. Apr 2008;16(2):132-143. [doi: 10.1037/1064-1297.16.2.132] [Medline: 18489017]

**46.** Tiburcio M, Lara MA, Martinez N, Fernandez M, Aguilar A. Web-Based Intervention to Reduce Substance Abuse and Depression: A Three Arm Randomized Trial in Mexico. Subst Use Misuse. Nov 10 2018;53(13):2220-2231. [doi: 10.1080/10826084.2018.1467452] [Medline: 29768070]

**47.** Moore BA, Buono FD, Lloyd DP, Printz DMB, Fiellin DA, Barry DT. A randomized clinical trial of the Recovery Line among methadone treatment patients with ongoing illicit drug use. J Subst Abuse Treat. Feb 2019;97:68-74. [doi: 10.1016/j.jsat.2018.11.011] [Medline: 30577901]

**50.** Kelpin SS, Parlier-Ahmad AB, Jallo N, Carroll K, Svikis DS. A pilot randomized trial of CBT4CBT for women in residential treatment for substance use disorders. J Subst Abuse Treat. Jan 2022;132:108622. [doi: 10.1016/j.jsat.2021.108622] [Medline: 34538690]

**51.** Campbell ANC, Rieckmann T, Pavlicova M, Choo TH, Molina K, McDonell M, et al. Culturally tailored digital therapeutic for substance use disorders with urban Indigenous people in the United States: A randomized controlled study. J Subst Use Addict Treat. Dec 2023;155:209159. [doi: 10.1016/j.josat.2023.209159] [Medline: 37690525]

**53.** Wilde JA, Zawislak K, Sawyer-Morris G, Hulsey J, Molfenter T, Taxman FS. The adoption and sustainability of digital therapeutics in justice systems: A pilot feasibility study. Int J Drug Policy. Jun 2023;116:104024. [doi: 10.1016/j.drugpo.2023.104024] [Medline: 37086698]

**54.** Xiong X, Braun S, Stitzer M, Luderer H, Shafai G, Hare B, et al. Evaluation of real-world outcomes associated with use of a prescription digital therapeutic to treat substance use disorders. Am J Addict. Jan 2023;32(1):24-31. [doi: 10.1111/ajad.13346] [Medline: 36264211]

**55.** Garland EL, Recasens M, Taple BJ, Donaldson GW, Weisberg RB. Virtual reality-based Mindfulness-Oriented Recovery Enhancement (MORE-VR) as an adjunct to

medications for opioid use disorder: a Phase 1 trial. Ann Med. Dec 2024;56(1):2392870. [doi: 10.1080/07853890.2024.2392870] [Medline: 39172534]

**56.** Coronado-Montoya S, Abdel-Baki A, Bodson-Clermont P, Boucher-Roy D, Cote J, Crocker CE, et al. A pilot randomized controlled trial of a digital cannabis harm reduction intervention for young adults with first-episode psychosis who use cannabis. Psychiatry Res. Aug 2025;350:116553. [doi: 10.1016/j.psychres.2025.116553] [Medline: 40450961]

**57.** Downie D, Patel A, Corman M, de Oliveira C, Jain E, Patterson M, et al. Computer-based training for cognitive behavioral therapy (CBT4CBT): A mixed methods investigation. Digit Health. Jan-Dec 2025;11:20552076251360919. [doi: 10.1177/20552076251360919] [Medline: 40688578]

**59.** Patel A, Corman M, de Oliveira C, Mason J, Some N, Downie D, et al. Computer-Based Training for Cognitive Behavioural Therapy for Substance Use Disorder: A Randomized Controlled Trial Including Quantitative and Qualitative Health and Economic Outcomes: Formation informatisee pour la therapie cognitivo-comportementale pour les troubles lies a l'usage de substances : un essai randomise controle y compris les resultats quantitatifs et qualitatifs en matiere de sante et d'economie. Can J Psychiatry. Jan 2025;70(1):9-20. [doi: 10.1177/07067437241255100] [Medline: 38783836]

**61.** Stidham Ba J, Jon-Emefieh Ba C, Carrano Ph DJ, Wenzel Ph DK, Fishman Md M. Characteristics of mHealth therapy app engagement by young adults with OUD. J Addict Dis. Jul-Sep 2025;43(3):201-206. [doi: 10.1080/10550887.2024.2363027] [Medline: 38914005]

**62.** Zhang X, Hardcastle V. Digital Contingency Management for Substance Use Disorder Treatment: 12-Month Quasi-Experimental Design. JMIR Ment Health. Sep 2 2025;12:e73617. [doi: 10.2196/73617] [Medline: 40896835]

**63.** Cardoso Sao Mateus C, Fletcher J, Rash C, DeFulio A. A retrospective comparison of two state-funded smartphone-based contingency management programs with different incentives. Drug Alcohol Depend. Aug 1 2026;285:113205. [doi: 10.1016/j.drugalcdep.2026.113205] [Medline: 42184588]

**64.** Kawasaki S, Lehman E, Kong L, Spangler A, Aydingolo N, Nunes E. A Digital Therapeutic Mobile App, ReSET-O, as Adjunct to Medication Treatment for Opioid Use Disorder: A Randomized Clinical Trial at an Opioid Treatment Program. J Addict Med. Mar 16 2026 [doi: 10.1097/ADM.0000000000001682] [Medline: 41833956]

**66.** Elison S, Jones A, Ward J, Davies G, Dugdale S. Examining effectiveness of tailorable computer-assisted therapy programmes for substance misuse: Programme usage and clinical outcomes data from Breaking Free Online. Addict Behav. Nov 2017;74:140-147. [doi: 10.1016/j.addbeh.2017.05.028] [Medline: 28645092]

**67.** Elison-Davies S, Martens K, Yau C, Davies G, Ward J. Associations between baseline opioid use disorder severity, mental health and biopsychosocial functioning, with clinical responses to computer-assisted therapy treatment. Am J Drug Alcohol Abuse. May 4 2021;47(3):360-372. [doi: 10.1080/00952990.2020.1861618] [Medline: 33428458]

**68.** Gunther F, Wong D, Elison-Davies S, Yau C. Identifying factors associated with user retention and outcomes of a digital intervention for substance use disorder: a retrospective analysis of real-world data. JAMIA Open. Oct 2023;6(3):ooad072. [doi: 10.1093/jamiaopen/ooad072] [Medline: 37663407]

**71.** Liang D, Han H, Du J, Zhao M, Hser YI. A pilot study of a smartphone application supporting recovery from drug addiction. J Subst Abuse Treat. May 2018;88:51-58. [doi: 10.1016/j.jsat.2018.02.006] [Medline: 29606226]

**72.** Zhang L, Li N, Li Y, Zhang T, Li D, Liu Y, et al. Preliminary efficacy of a digital therapeutics smartphone application for methamphetamine use disorder: An experimental study. Front Psychiatry. 2022;13:1027695. [doi: 10.3389/fpsyt.2022.1027695] [Medline: 36339836]

**73.** Carroll KM, Ball SA, Martino S, Nich C, Babuscio TA, Nuro KF, et al. Computer-assisted delivery of cognitive-behavioral therapy for addiction: a randomized trial of CBT4CBT. Am J Psychiatry. Jul 2008;165(7):881-888. [doi: 10.1176/appi.ajp.2008.07111835] [Medline: 18450927]

**74.** Budney AJ, Stanger C, Tilford JM, Scherer EB, Brown PC, Li Z, et al. Computer-assisted behavioral therapy and contingency management for cannabis use disorder. Psychol Addict Behav. Sep 2015;29(3):501-511. [doi: 10.1037/adb0000078] [Medline: 25938629]

**75.** DeFulio A, Rzeszutek MJ, Furgeson J, Ryan S, Rezania S. A smartphone-smartcard platform for contingency management in an inner-city substance use disorder outpatient program. J Subst Abuse Treat. Jan 2021;120:108188. [doi: 10.1016/j.jsat.2020.108188] [Medline: 33298295]
